# Patterns of whole-body muscle activations following vertical perturbations during standing and walking

**DOI:** 10.1186/s12984-021-00836-0

**Published:** 2021-05-06

**Authors:** Desiderio Cano Porras, Jesse V. Jacobs, Rivka Inzelberg, Yotam Bahat, Gabriel Zeilig, Meir Plotnik

**Affiliations:** 1 Center of Advanced Technologies in Rehabilitation, Sheba Medical Center, Ramat Gan, Israel; 2 Sackler Faculty of Medicine, Tel Aviv University, Tel Aviv, Israel; 3 Perception and Action in Complex Environments, Marie Curie International Training Network, European Union’s Horizons 2020 Research and Innovation Program, Brussels, Belgium; 4 Brightlands Institute for Smart Society-BISS, Maastricht University, Maastricht, The Netherlands; 5 Rehabilitation and Movement Science, University of Vermont, Burlington, VT USA; 6 Sagol School of Neuroscience, Tel Aviv University, Tel Aviv, Israel; 7 Department of Neurology and Neurosurgery, Sackler Faculty of Medicine, Tel Aviv University, Tel Aviv, Israel; 8 Department of Applied Mathematics and Computer Science, The Weizmann Institute of Science, Rehovot, Israel; 9 Department of Neurological Rehabilitation, Sheba Medical Center, Tel HaShomer, Ramat Gan, Israel; 10 Department of Physical and Rehabilitation Medicine, Sackler Faculty of Medicine, Tel Aviv University, Tel Aviv, Israel; 11 Department of Physiology and Pharmacology, Sackler Faculty of Medicine, Tel Aviv University, Tel Aviv, Israel

**Keywords:** Virtual reality, Balance, Gait, Perturbations, Postural control, EMG

## Abstract

**Background:**

Falls commonly occur due to losses of balance associated with vertical body movements (e.g. reacting to uneven ground, street curbs). Research, however, has focused on horizontal perturbations, such as forward and backward translations of the standing surface. This study describes and compares muscle activation patterns following vertical and horizontal perturbations during standing and walking, and investigates the role of vision during standing postural responses.

**Methods:**

Fourteen healthy participants (ten males; 27±4 years-old) responded to downward, upward, forward, and backward perturbations while standing and walking in a virtual reality (VR) facility containing a moveable platform with an embedded treadmill; participants were also exposed to visual perturbations in which only the virtual scenery moved. We collected bilateral surface electromyography (EMG) signals from 8 muscles (tibialis anterior, rectus femoris, rectus abdominis, external oblique, gastrocnemius, biceps femoris, paraspinals, deltoids). Parameters included onset latency, duration of activation, and activation magnitude. Standing perturbations comprised dynamic-camera (congruent), static-camera (incongruent) and eyes-closed sensory conditions. ANOVAs were used to compare the effects of perturbation direction and sensory condition across muscles.

**Results:**

Vertical perturbations induced longer onset latencies and shorter durations of activation with lower activation magnitudes in comparison to horizontal perturbations (p<0.0001). Downward perturbations while standing generated earlier activation of anterior muscles to facilitate flexion (for example, p=0.0005 and p=0.0021 when comparing the early activators, rectus femoris and tibialis anterior, to a late activator, the paraspinals), whereas upward perturbations generated earlier activation of posterior muscles to facilitate extension (for example, p<0.0001 and p=0.0004, when comparing the early activators, biceps femoris and gastrocnemius, to a late activator, the rectus abdominis). Static-camera conditions induced longer onset latencies (p=0.0085 and p<0.0001 compared to eyes-closed and dynamic-camera conditions, respectively), whereas eyes-closed conditions induced longer durations of activation (p=0.0001 and p=0.0008 compared to static-camera and dynamic-camera, respectively) and larger activation magnitudes. During walking, downward perturbations promptly activated contralateral trunk and deltoid muscles (e.g., p=0.0036 for contralateral deltoid versus a late activator, the ipsilateral tibialis anterior), and upward perturbations triggered early activation of trunk flexors (e.g., p=0.0308 for contralateral rectus abdominis versus a late activator, the ipsilateral gastrocnemius). Visual perturbations elicited muscle activation in 67.7% of trials.

**Conclusion:**

Our results demonstrate that vertical (vs. horizontal) perturbations generate unique balance-correcting muscle activations, which were consistent with counteracting vertical body extension induced by downward perturbations and vertical body flexion induced by upward perturbations. Availability of visual input appears to affect response efficiency, and incongruent visual input can adversely affect response triggering. Our findings have clinical implications for the design of robotic exoskeletons (to ensure user safety in dynamic balance environments) and for perturbation-based balance and gait rehabilitation.

**Supplementary Information:**

The online version contains supplementary material available at 10.1186/s12984-021-00836-0.

## Introduction

Losses of balance associated with vertical maneuvers are common in daily life, such as when experiencing mis-steps on uneven ground or reacting to unexpected steps or street curbs [1–4]. Despite their commonality, the research on balance recovery mechanisms heavily focuses on horizontal perturbations [5–7]. The literature would benefit from a more mechanistic understanding of balance recovery in response to vertical perturbations in order to inform rehabilitation strategy or to support programming for assistive devices [8–11]. Losing balance involves vertical as well as horizontal and rotational displacements. Vertical body motion, in particular, affects both the body height and vertical body loading [12]; for instance, mis-stepping stairs or standing in an unstable moving bus or subway [13]. Because strategies of balance recovery lead to task-specific, oriented muscular arrangements that define the resulting postural adjustments [6, 12], biomechanical strategies of balance recovery must account for the vertical dislocation of the body as well. Therefore, in support of these goals, this study compares the muscle activation patterns induced by vertical versus horizontal perturbations. Further, because vertical losses of balance appear related to mis-stepping on non-uniform surfaces or to maintaining standing balance in enclosed transportation, we also explore the role of vision and incongruent visual input on these responses.

Sudden, unexpected physical perturbations exerted over the human body can be a destabilizing force that compromises balance [6, 14]. For restoring balance after sudden physical perturbations, the central nervous system engages in rapid postural responses of contextually relevant muscle activation patterns [6, 7, 15–18]. These responses comprise complex patterns of muscle activation that progressively develop via supraspinal control [19] and are dependent on multisensory feedback [5, 17, 20]. Given the complex and context-dependent control of postural responses to extrinsic perturbations, it is important to understand the muscle response patterns to vertical perturbations.

After horizontal perturbations, the strategy adopted by healthy adults often employs a distal-to-proximal, reciprocal muscle activation pattern; for example, anterior muscles such as the tibialis anterior, rectus femoris and rectus abdominis are primarily activated after anterior surface translations to correct an induced backward fall, and posterior muscles such as gastrocnemius, biceps femoris, and erector spinae are primarily activated after posterior surface translations to correct an induced forward fall [15–17, 21]. Fewer studies evaluated postural responses to vertical perturbations (in comparison to horizontal perturbations) during standing [12, 22, 23] or walking [24–26]. The aforementioned studies, however, focused on stretch reflexes from leg muscles, not accounting for the coordinated, progressive, multi-segmental responses necessary to maintain functional balance [27]. The present study thus sought to more comprehensively elucidate leg-trunk-shoulder muscle activation patterns that characterize balance-correcting responses to vertical perturbations during standing and walking.

We hypothesized that muscle activation patterns in response to vertical perturbations will differ from those in response to horizontal perturbations, because vertical perturbations generate unique challenges to equilibrium [6, 12, 15, 28, 29]. Particularly, we predicted initial activation of trunk flexors/extensors in maintaining balance after vertical perturbations, given the primary role of the trunk as stabilizer/prime-mover for restoring balance [5, 6, 16, 30] (for a detailed rationale of a priori predictions and hypotheses, see Additional file 1). In addition, because vertical visual scenes influence the apparent direction of gravity [31], we expect that visual conditions will modulate postural responses by adjusting the body against expected gravitational forces following vertical perturbations, and by activating muscles opposing a potential fall following horizontal perturbations. We further hypothesized that the role of vision would differ for vertical versus horizontal perturbations due to the critical role of vision in maintaining equilibrium [32–34], partly because visual perturbations can signal gravitational changes as well as positional changes [31, 35]. Last, because postural responses progressively adapt to environmental visual cues, and there is an attenuation of initial activity when visual cues are inconsistent with other sensory cues [36], we anticipate that incongruent (and absence of) vision will lead to longer latency, longer duration, and increased magnitude of activation.

## Methods

### Participants

Fourteen young, healthy adults (mean age ± SD: 27 ± 4 years; BMI: 23.8 ± 2.6 kg/m^2^; 10 males, 4 females) participated in this study. None of the participants had sensory, motor or cognitive limitations that could potentially affect balance or gait. All participants were able to follow instructions and gave written informed consent before being enrolled in the study. The Institutional Review Board for Ethics in Human Studies at the Sheba Medical Center, Israel, approved the experimental protocol.

### Apparatus

**Fig. 1:**
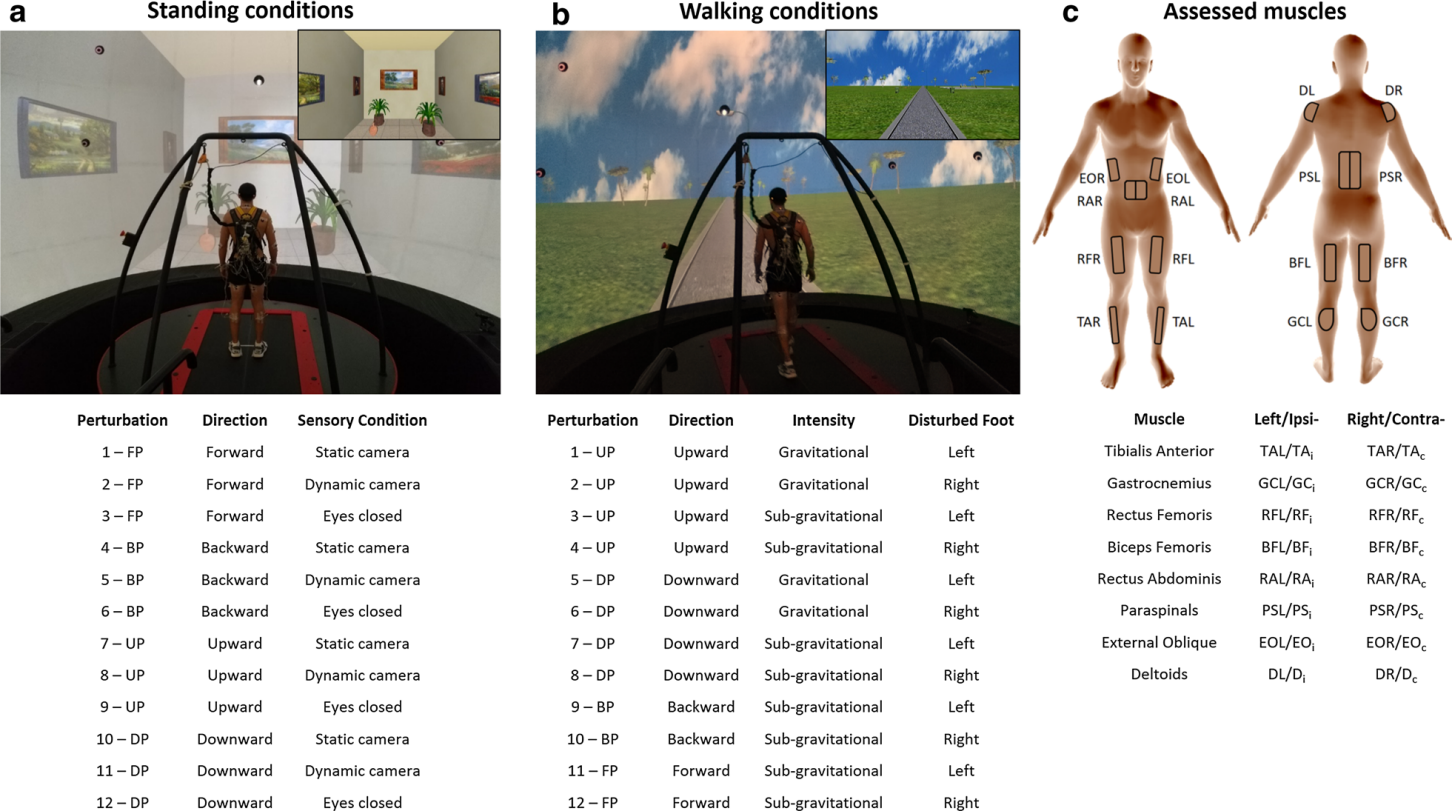
Apparatus and experimental conditions. **a** Virtual visual scenery of a simulated room used for both physical and visual perturbations while standing. Objects and walls in the virtual room provided depth cues that were manipulated in relation to the physical actions of the platform and participant. There were four perturbation directions (forward, backward, upward, and downward; FP, BP, UP, DP, respectively) and three sensory conditions: static-camera, dynamic-camera, and eyes closed. **b** Virtual visual scenery used during walking conditions projected a moving road on a large 360° dome-shaped screen. There were four perturbation directions, and perturbations occurred either during left or right foot contact. **c** Depiction of the 16 muscles assessed. Ipsilateral muscles (sub-index “i”) refer to those recorded from the perturbed stance foot during walking conditions (contralateral denoted by sub-index “c”); i.e., the layout in panel C depicts indexing relevant to perturbation induced during left foot stance. The electrodes’ positions were determined according to SENIAM guidelines. *TA* tibialis anterior, *GC* gastrocnemius lateralis, *RF* rectus femoris, *BF* biceps femoris, *RA* rectus abdominis, *PS* paraspinal, *EO* external oblique, *D* deltoid medial, *L* left, *R* right

Experiments were conducted with a fully immersive virtual reality system (CAREN High End, Motek Medical, The Netherlands; Fig. 1), containing a moveable platform with six degrees of freedom [37]. A motion capture system (Vicon, Oxford, UK) concurrently tracked the three-dimensional coordinates of 41 passive-reflective markers affixed to the body of each participant with a sampling rate of 120 Hz and spatial accuracy of 1 mm. The implemented marker setup followed Vicon’s ‘HumanRTKM’ model [38]. The platform included two force plates and contained an embedded treadmill that operated in self-paced mode, allowing participants to adjust treadmill speed to preferred walking speed [39]. The algorithm operating the self-paced mode functions by biofeedback based on the markers positioned around the waist. Forward movements of the body increase treadmill speed, whereas backward movements of the body slow down treadmill speed. An electromyography (EMG) system (ANT Neuro, Hengelo, The Netherlands) captured bipolar electrical activity of muscle activation with a sampling frequency of 1024 Hz bilaterally from the tibialis anterior, gastrocnemius lateralis, rectus femoris, biceps femoris, rectus abdominis, paraspinals, external oblique and medial deltoid (Fig. 1c).

### Visual scenery

Standing and walking conditions incorporated two different visual scenes (Fig. 1a, b). In standing conditions, we implemented a simulated room with objects such as paintings on the wall and plants on the floor. Objects provide depth cues and influence motion perception (e.g., via lines of depth perspective). In walking conditions, visual scenes simulated walking on an asphalt road in a park, with a brick wall ending at the horizon and greenery adjacent to the wall. Scenes were modeled in three dimensions with specialized software (Autodesk XSI). Textures were created and modified with Adobe Photoshop. Custom software (D-Flow, Motek Medical, The Netherlands) was used for programing, integration, and projection, as well as for displacing the platform and activating the treadmill in a synchronized manner with the EMG recording system. We placed a virtual camera in the virtual world, representing both the center of the lab and the center of the moveable platform. During the experimental walking conditions, the visual scene advanced in the sagittal plane (i.e., visual flow) at a speed synchronous with the speed of the treadmill. Accordingly, to provide the effect of actually walking on the park road, the entire virtual world (i.e. projected environment) was programmed to move congruently around the virtual camera. The visual scenes were projected on a 360° dome-shaped screen (six meters in diameter) by eight video projectors. Projector resolution was 1400x1050 pixels, and participant viewing distance was 3 m.

### Experimental procedures

A familiarization period for mastering the self-paced mode of the treadmill (10–15 min) followed the calibration of EMG and motion capture systems. After the familiarization period, trials included ‘visual’, ‘standing’ and ‘walking’ perturbations. During visual perturbations, there is no physical movement of the platform where participants stand (only the visual scenes move); because of this, we differentiate visual from *physical* (i.e. standing, walking) perturbations.

Participants were first exposed to *visual perturbations* (the virtual room moves a distance corresponding to a platform movement of 14 cm in 350 ms while the participant is standing in an unmoving physical environment). Visual perturbations were downward, upward, forward and backward. Each perturbation direction repeated three times in random order, totaling 12 visual perturbations. The person was standing in the static virtual room and the first perturbation occurred after 45 s; subsequent perturbations occurred according to a random epoch of 5–10 seconds. The implementation of the random epoch and randomized directions aimed to reduce the predictability of the visual perturbations in both timing and direction, but it does not necessarily mitigate the expectation of an impending perturbation.

Physical perturbations followed visual perturbations. Participants were exposed to 12 types of perturbations during standing and 12 types of perturbations during walking (Fig. 1a, b). Each type of perturbation was repeated three times, totaling 36 perturbations (i.e., 72 physical perturbations combined across standing and walking conditions). Both standing and walking perturbations were divided into three trial blocks. Each trial block included 12 perturbations taking place in random order (i.e. three blocks for standing and three blocks for walking). To minimize learning effects, before starting the research experiments, we randomized for all participants to start with either a standing or walking trial block. Trial blocks were then alternated between standing and walking until completing all six blocks. Within each single block, the type of perturbation was also randomized (out of 12 possibilities; see Fig. 1).

Walking was performed in self-paced mode. Except in one occasion, however, due to technical constraints (for one participant) the treadmill was limited to a maximum walking speed of 1.4m/s. The dataset generated was included in analysis because, despite the imposed limit on walking speed, on this occasion, the participant exhibited similar balance performance to other occasions (within the same participant), and performance was consistent to that of most participants. All trial blocks were completed in a single session, and participants were offered a resting period in all intervals between trial blocks.

### Standing perturbations (see Fig. 1a)

Perturbations were downward, upward, forward, and backward, each within three sensory conditions: dynamic-camera, static-camera, and eyes-closed. In dynamic-camera conditions, the virtual room moved according to the physical displacement of the platform, whereas in static-camera conditions, the virtual room remained static, thus providing a sense of sensory conflict or incongruence. During eyes-closed conditions, a pre-recorded audio message asked the person to close their eyes, which was accompanied by the virtual environment being turned off; and then after the perturbation occurred, a pre-recorded audio message asked the person to open their eyes and the virtual environment was turned on. The experimenter periodically confirmed via direct observation that the participant was complying with the recorded audio commands. No ‘ignoring’ behavior was noted.

Time between perturbations in 'standing conditions' was between 8 and 15 s. We used upward perturbations to balance participant expectations to downward perturbations (i.e. to avoid expecting downward perturbations in larger proportion), and horizontal perturbations for comparison due to the more-developed literature for horizontal perturbations. Perturbations were displacements of the moveable platform integrated into the VR system. The displacement was 12 cm, with a duration of 1000 ms for each direction. These parameters were chosen to facilitate feet-in-place responses rather than inducing stepping responses. Participants were asked to maintain an upright position with the feet within marked boundaries, defined by the initial positioning of their feet (i.e. stance width) when asked to stand comfortably. Perturbation intensity was selected to allow participants to maintain balance without stepping [40]. Participants were able to maintain balance in response to the perturbations, thus it was not needed to repeat trials.

### Walking perturbations (see Fig. 1b and Additional file 2: Video S1)

There were four perturbation directions: downward, upward, forward and backward. All directions included perturbations of sub-gravitational intensity (i.e. a platform displacement of 20 cm occurring in 300 ms). In addition, vertical perturbations (downward, upward) included approximated gravitational intensities (i.e. 20 cm in 202 ms). In total, there were 12 types of walking perturbations (Fig. 1b). In this study, however, we focus on perturbations with sub-gravitational intensities for comparison of the four directions. Perturbations were displacements of the moveable platform occurring during either left- or right-foot contact of early stance [25, 26]. A real-time algorithm identified foot contact based on a combination of the vertical coordinates of heel markers and the pressure force on the platform. The first perturbation was triggered either after attaining steady-state velocity (SSV) or after one minute (without attaining SSV), and a random epoch (<10 seconds) was added for triggering subsequent perturbations to reduce the predictability of the perturbations in both timing and direction, which limits a participant’s ability to pre-plan responses or generate anticipatory responses. SSV was defined as walking at least 30 seconds with a 12-second consecutive period in which the coefficient of variance of walking speed was less than 2%, either after initiation of a trial block or since the previous perturbation occurred.

### Analysis of EMG activity (Fig. 2)

**Fig. 2:**
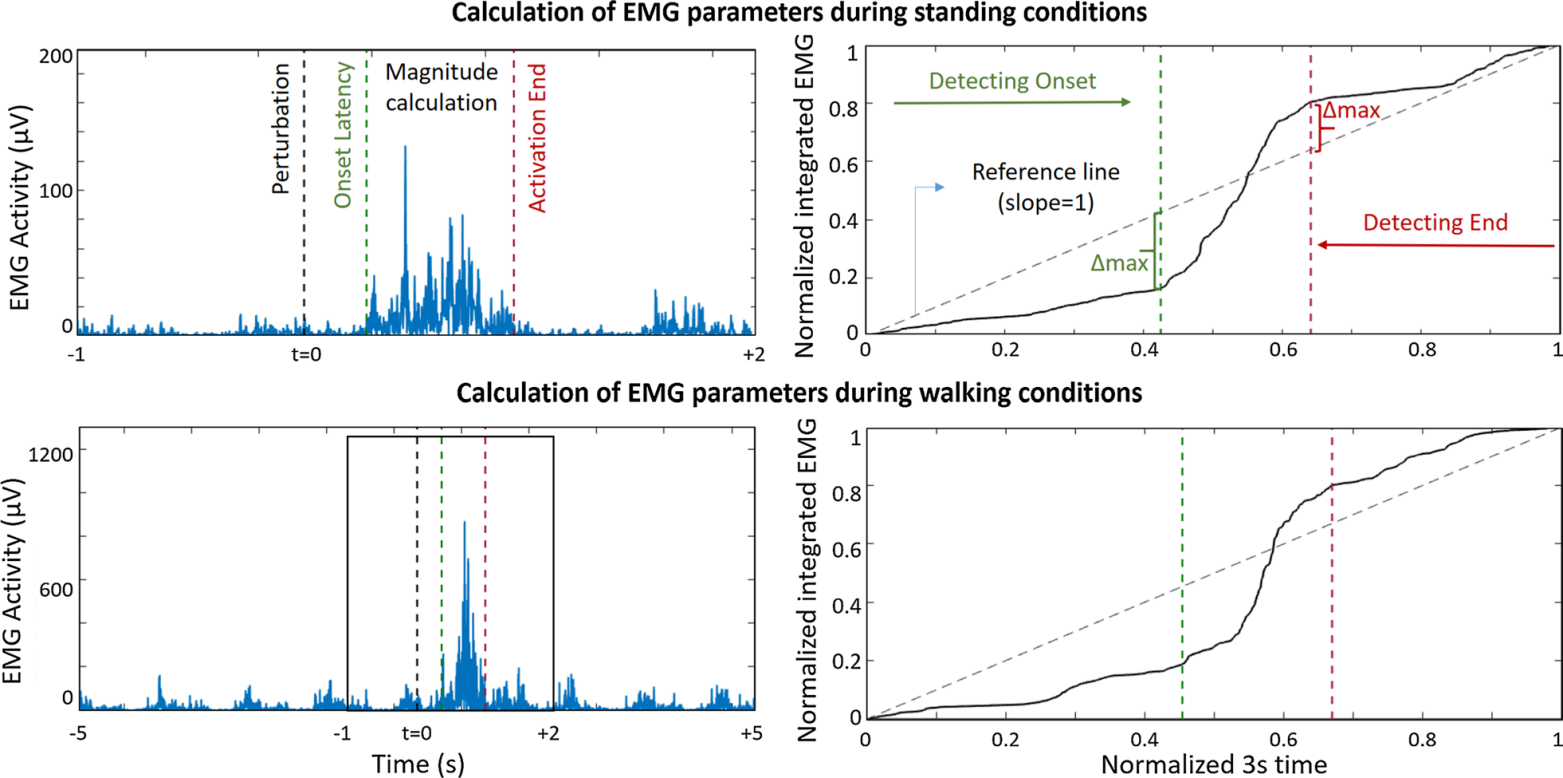
Calculation of electromyography (EMG) parameters during standing (upper plot) and walking (lower plot). The left column shows the filtered and rectified EMG activity with the calculated EMG parameters. The EMG signal is integrated and normalized in amplitude and time (right column) only for calculating “onset latency” and “activation end”. The detection of onset latency is defined as the maximum delta between a unitary, reference line (diagonal dashed line) and the integrated, normalized EMG signal. A similar approach, after inverting the signal, is used to identify the end of activation. Duration of activation is determined as the period between onset latency and the end of activation. For estimating activation magnitude, we calculated the area under the curve in the duration of activation period, on the filtered and rectified original EMG signal

EMG signals were filtered (finite impulse response band-pass of 20–400 Hz) and full-wave rectified. Calculated EMG parameters were onset latency, duration of activation, and activation magnitude. The calculation of EMG parameters was based on a three-second window: one second before (baseline) and two seconds after perturbation onset. EMG signals were then integrated and normalized within each window, i.e. normalized so that the integrated EMG value and the time for analysis had a final value of 1. The latter process is intended for the purpose of identifying burst onset and burst offset, and not for calculation of activation magnitude. Onset latency was defined as the period between perturbation onset time and the onset of EMG activity beyond baseline levels. The method for calculating onset latency has been previously described in detail [41]. In summary, onset latency represents the largest distance between the normalized, integrated EMG signal and a unitary line serving as reference. The algorithm focuses on identifying the early burst of EMG activity occurring after perturbations. When the rate of growth in the normalized, integrated EMG (for each three-second window of analysis) identifies an onset latency before perturbation time, there is an indication that the EMG activity did not increase after perturbation. We marked those cases as “no response”. The same method was used to calculate the duration of activation. For this, the three-second window of the EMG signal was inverted in order to identify the initial (i.e. the last) part of the EMG burst. Duration of activation was defined as the period elapsing between the onset and the end of the EMG burst. We used numerical integration (i.e. trapezoidal method) on the identified period of EMG activation for the calculation of activation magnitude (i.e. over the filtered and rectified EMG signal, between “onset latency” and “activation end”, see Fig. 2). Because the evaluated burst period led to unequal durations, we calculated magnitude based on numerical integration for every single perturbation to obtain average magnitude during burst activity. In each type of perturbation, we averaged the values of the three repetitions for each participant, without including in the average those cases marked as “no response”. No rescaling or normalization was employed to calculate activation magnitude due to the within-subject, intra-session nature of this study’s analysis.

### Analysis of extrapolated center of mass

In standing conditions, we computed 95% confidence ellipses for extrapolated center of mass (COM_x_) [42, 43], both during baseline (i.e. six seconds before perturbation onset) and for 5 seconds after perturbation onset (period defined after an overall visual inspection of commonly occurring effects). COM_x_ is an extrapolation of the center of mass trajectory in the direction of its velocity, and the COM_x_ position within the boundaries of the base of support provides a measure of postural stability [42, 43]. From each ellipse, we evaluated area, major and minor axis length, and orientation angle. Ellipse calculations followed previous methodologies [44].

### Statistical analyses

The data are expressed as mean ± standard deviation unless otherwise stated. The Shapiro-Wilk calculation was used to test the hypothesis of normal distributions. For standing conditions, we computed a three-way analysis of variance (3 sensory conditions × 4 perturbation directions × 16 muscles) for two of the EMG parameters (onset latency, duration of activation). Due to the sensibility of the electrode position and the within-session nature of the experiment, for magnitude we applied a two-way analysis of variance per each muscle. For walking conditions, muscles were arranged as ipsilateral and contralateral muscles according to the side of stance-foot perturbation. We computed a three-way analysis of variance for onset latency and duration of activation (4 perturbation directions × 2 perturbation sides × muscle); for magnitude we computed a two-way analysis of variance for each muscle. For post hoc comparisons, we used the Fisher’s least significant difference procedure. For comparison between baseline and after-perturbation values, we utilized two-tailed t-tests. The level of significance was set to 5% (p < 0.05). Statistical procedures were implemented with a numerical computing environment (Matlab; The Mathworks, Natick, MA).

## Results

### Muscle activation in response to perturbations during standing (Fig. 3)

**Fig. 3:**
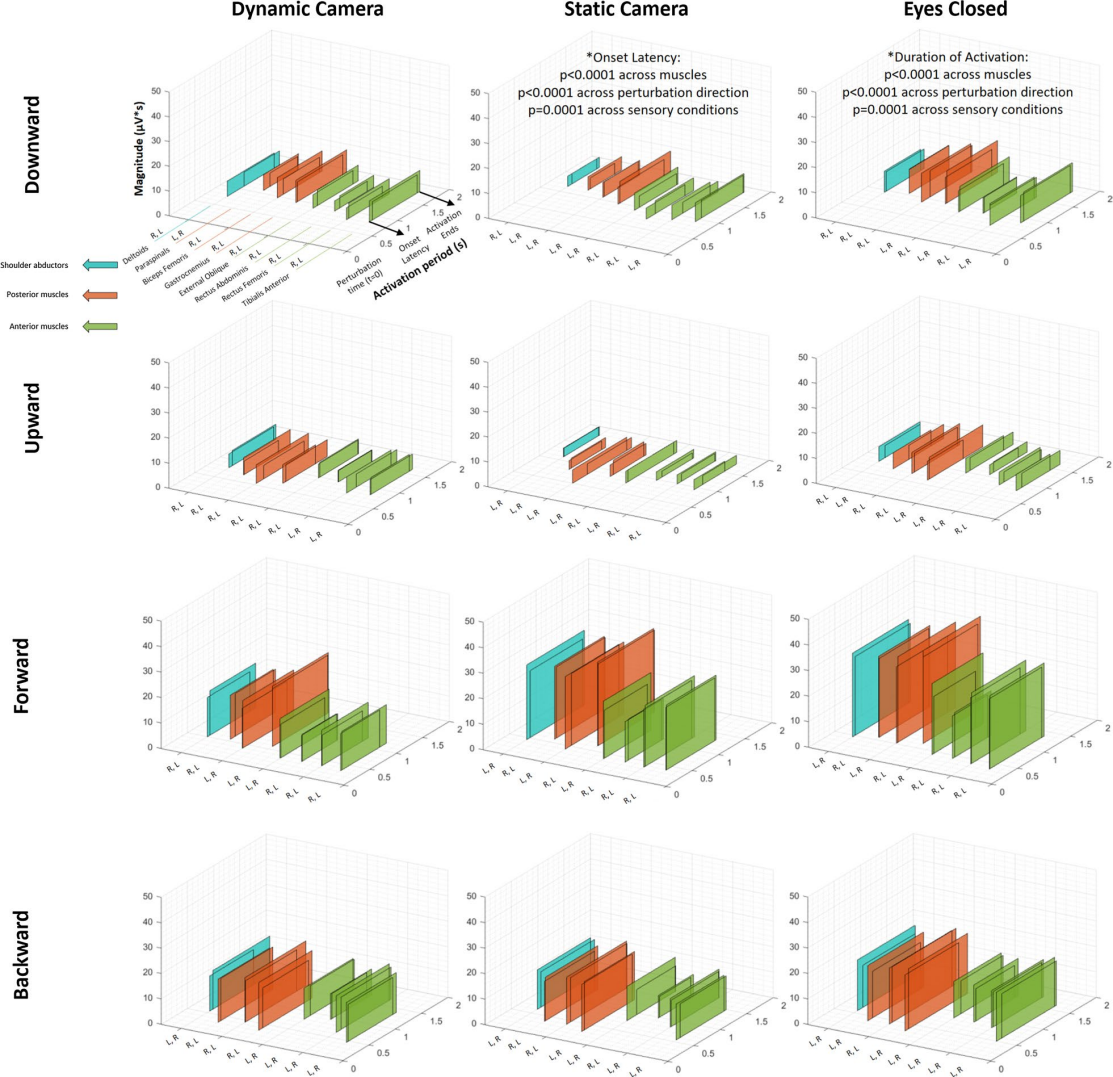
Patterns of muscle activation following perturbations while standing. Description of muscle activation patterns arranged by 3-D rectangles representing groups of shoulder abductors (blue), posterior muscles (orange) and anterior muscles (green). Figures show average values (from all participants) of onset latency and duration of activation (“Activation period” axis), and activation magnitude (vertical axis). (Additional file 3: Table S1) shows values of EMG parameters during standing conditions. The overlapping rectangles depict left (L) and right (R) body sides. The letters indicate the shorter and longer onset latency, respectively. *: p-values observed within the main factors characterizing the ANOVA statistical analysis (see text and Additional file 6 for more details)

Figure 3 illustrates the sequence and magnitude of muscle activations (color-coded) in response to physical perturbations during the standing conditions. The figure comprises data on EMG onset latency, duration and magnitude of each muscle activation seen during all standing experimental conditions. Complementing this figure is Additional file 3: Table S1, which details the corresponding numeric values. In addition, Additional file 6 provides the statistical output from the analyses of onset latency and duration of activation during standing conditions.

#### Description of muscle onset patterns of vertical perturbations

During standing conditions, downward perturbations led to a shorter onset latency of anterior muscles (see Additional file 3: Table S1, Fig. 3) (earliest activation was observed at the rectus femoris; e.g. 0.66 ± 0.32 s, right-side muscle after eyes-closed conditions) when compared to posterior muscles (latest activation was observed at the paraspinals; e.g. 1.03 ± 0.22 s, right-side muscle after eyes-closed conditions). For instance, the pattern was observed in all comparisons between left and right paraspinals vs. all anterior muscles (e.g. p = 0.0053 and p = 0.0004 when compared to left tibialis anterior), with the only exception being left paraspinal vs. left rectus abdominis (p = 0.0579); the pattern was also evident in the comparisons between left biceps femoris vs. left tibialis anterior (p = 0.0162), left and right rectus femoris (p = 0.0021 and p < 0.0001, respectively), right rectus abdominis (p = 0.0063) and left and right external oblique (p = 0.0317 and p = 0.0241, respectively); and for right biceps femoris vs. right rectus femoris (p = 0.0025) (all comparisons based on the computation of marginal means for each combination of muscle and perturbation direction, independent of the factor for sensory condition).

In contrast, upward perturbations triggered earlier onset latencies of posterior muscles, marked by earliest activation at the biceps femoris (see Additional file 3: Table S1, Fig. 3) (e.g. 0.50 ± 0.39s, left-side muscle following static-camera conditions) and latest activation at the rectus abdominis (e.g. 1.08 ± 0.49s, right-side muscle following static-camera conditions). For instance, both (left and right) gastrocnemius and biceps femoris had an onset latency significantly earlier than the anterior muscles (e.g. p = 0.0026 for right gastrocnemius; and p=0.0001 for right biceps femoris, respectively, compared to right rectus femoris); the only exceptions were left gastrocnemius vs. right tibialis anterior (p = 0.1352) and right gastrocnemius vs. right tibialis anterior (p = 0.1061). Finally, the onset latency of lower-leg antagonist muscles (i.e. tibialis anterior and gastrocnemius) were not statistically different to each other following vertical perturbations, except that the left tibialis anterior was activated earlier than left and right gastrocnemius after upward perturbations (p = 0.0070 and p = 0.0048, respectively; see Fig. 3).

#### Muscle activation—comparison of vertical and horizontal perturbations

Forward perturbations led to earlier onset latencies of anterior muscles, whereas posterior muscles were the first to respond after backward perturbations; similar to downward and upward perturbations, respectively (see Additional file 3: Table S1, Fig. 3). Unlike vertical perturbations, during which proximal leg muscles activated first, forward and backward perturbations elicited initial activation of the distal leg muscles (tibialis anterior and gastrocnemius). For instance, following forward perturbations, left tibialis anterior activated earlier than left and right paraspinals (respectively p=0.0004 and p=0.0039). In turn, following backward perturbations, right gastrocnemius activated earlier than both left and right rectus abdominis and external oblique (p<0.0001 for each of the four comparisons).

Onset latencies were shorter during horizontal perturbations (fastest responses followed forward perturbations) in comparison to vertical perturbations (downward perturbations generated the most delayed responses); for instance, p<0.0001 in the comparison between downward and forward perturbations, independent of the factors for muscle and sensory condition.

Duration of activation was longer following horizontal perturbations in comparison to vertical perturbations, with backward perturbations eliciting the longest durations (p < 0.0001 in the comparisons of backward, forward perturbations vs. downward, upward perturbations, independent of the factors for muscle and sensory condition).

Magnitude of activation was usually larger for horizontal (forward and backward) perturbations when compared to vertical perturbations (downward and upward). The pattern was observed for all muscles. For example, magnitude of the right biceps femoris was larger for backward than for downward perturbations (p = 0.0016), and larger for backward than for upward perturbations (p < 0.0001).

#### Muscle activation—comparison of visual conditions during physical perturbations

Static-camera conditions led to longer onset latencies in comparison to both eyes-closed (p = 0.0085) and dynamic-camera conditions (p < 0.0001).

The eyes-closed condition led to longer durations of activation in comparison to static-camera (p < 0.0001) and dynamic-camera conditions (p = 0.0008).

Statistically different magnitudes were observed across the three sensory conditions. For instance, the eyes-closed condition elicited the largest magnitudes, which were significantly higher than in the dynamic-camera condition that elicited the lowest activation magnitudes. This pattern was evident for all muscles; e.g., for the left paraspinals, in the comparisons between eyes-closed vs. static camera (p = 0.0445) and vs. dynamic camera conditions (p = 0.0014). Exceptions to this pattern were the right rectus femoris (p = 0.0751, from ANOVA results within the sensory condition factor), right external oblique (p=0.085), and for both left and right rectus abdominis muscles (respectively, p = 0.0954 and p = 0.1206).

#### Extrapolated center of mass—description and comparison after vertical and horizontal perturbations

**Fig. 4:**
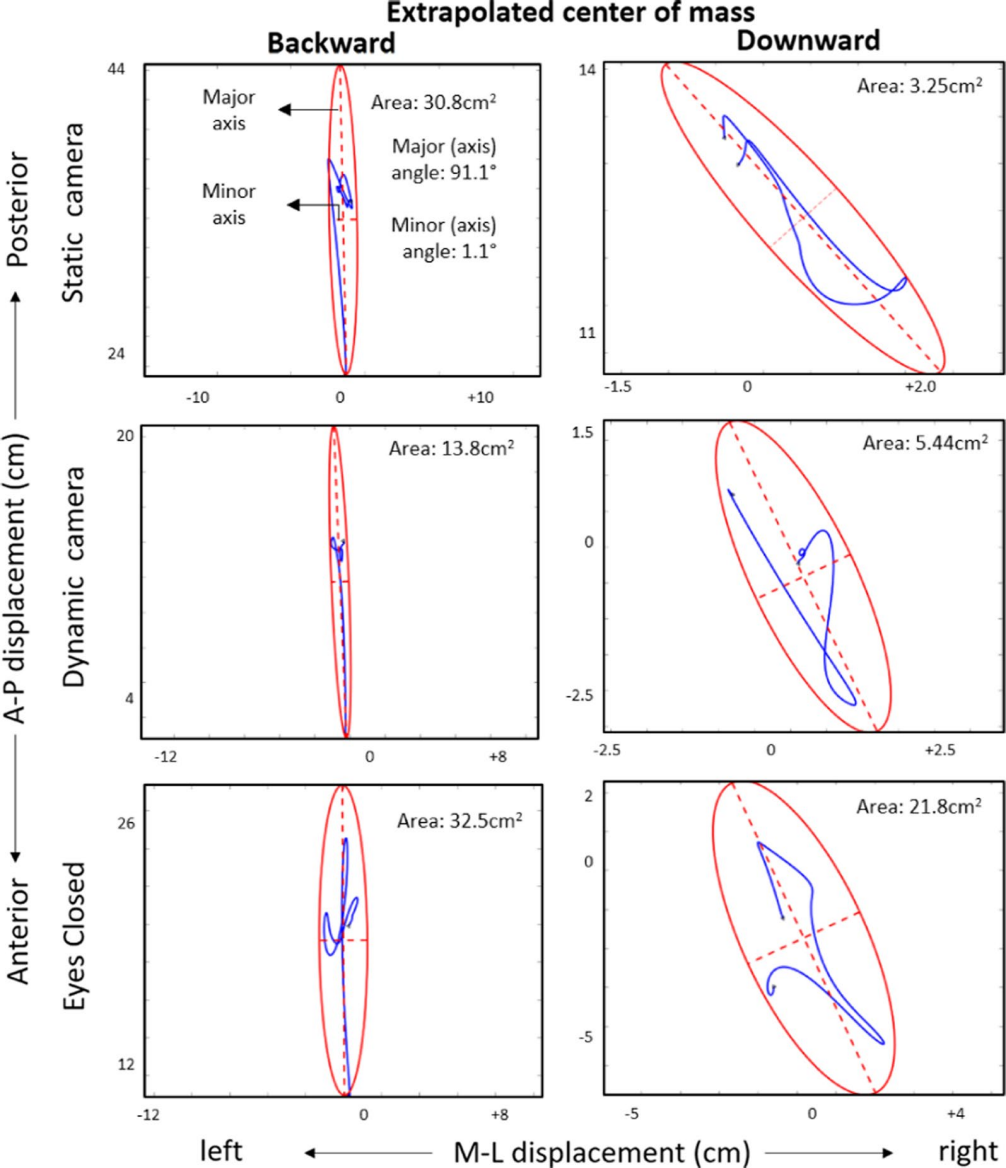
Ellipse fits for extrapolated center of mass (COM_x_) from two representative participants after downward and backward perturbations. Blue lines identify COM_x_ antero-posterior (A-P) and medio-lateral (M-L) displacements. 95% confidence ellipses appear in red. Characteristic minor and major axes are shown as dotted lines. Ellipse features following forward perturbations were similar to those after backward perturbations. Minor and major axis angles were near 0° and 90° respectively, suggesting a dominant anterior-posterior postural reaction

Major ellipse angles were significantly larger (over 90°) following both downward and upward perturbations, in comparison to both forward and backward perturbations (during which, angles were approximately 90°) (Fig. 4). Vertical perturbations generated larger minor ellipse angles (approximately 30°) in comparison to horizontal perturbations (approximately zero degrees) (p < 0.0001). Moreover, minor ellipse angles were greater in static-camera conditions in comparison to both dynamic-camera (p = 0.0318) and eyes-closed conditions (p = 0.0115).

The area of the extrapolated center of mass was significantly larger following forward perturbations when compared to the remaining three perturbation directions (p < 0.0001). Similar results were found for the major and minor axes of the ellipses. Backward perturbations elicited larger areas than both downward (p = 0.0004) and upward perturbations (p = 0.0003), and backward perturbations elicited larger major (but not minor) axes than both vertical perturbations (p < 0.0001).

#### Extrapolated center of mass—comparison of visual conditions and before vs. after perturbation

In the analysis of conditions prior to perturbation onset, we found that eyes-closed conditions generated larger major ellipse angles (near 90°) in comparison to both static-camera (p = 0.0100) and dynamic-camera conditions (p = 0.0084).

In the comparisons pre vs. post perturbation onset, we found that ellipse area, as well as major and minor axes increased after perturbation, which occurred for all combinations of sensory conditions and perturbation directions (e.g. respectively, p = 0.0002, p = 0.0001 and p < 0.0001 for downward perturbations in dynamic camera conditions). However, major and minor axis angles only exhibited significant changes for both downward and upward perturbations and not for horizontal perturbations (p < 0.05). For example, for upward perturbations with static camera, we observed changes in major (p = 0.0247) and minor angles (p = 0.0125); but for forward perturbations in dynamic camera no changes were observed in major (p = 0.3441) and minor angles (p = 0.4652). Importantly, major angles for dynamic- and static-camera conditions usually transitioned from ~ 60°–70° (before perturbation) to ~ 110°–120° (after perturbation), while major axis angles during the eyes-closed condition commonly exhibited a transition from ~ 90° to ~ 30°.

### Muscle activations in response to perturbations during walking (Fig. 5)

**Fig. 5:**
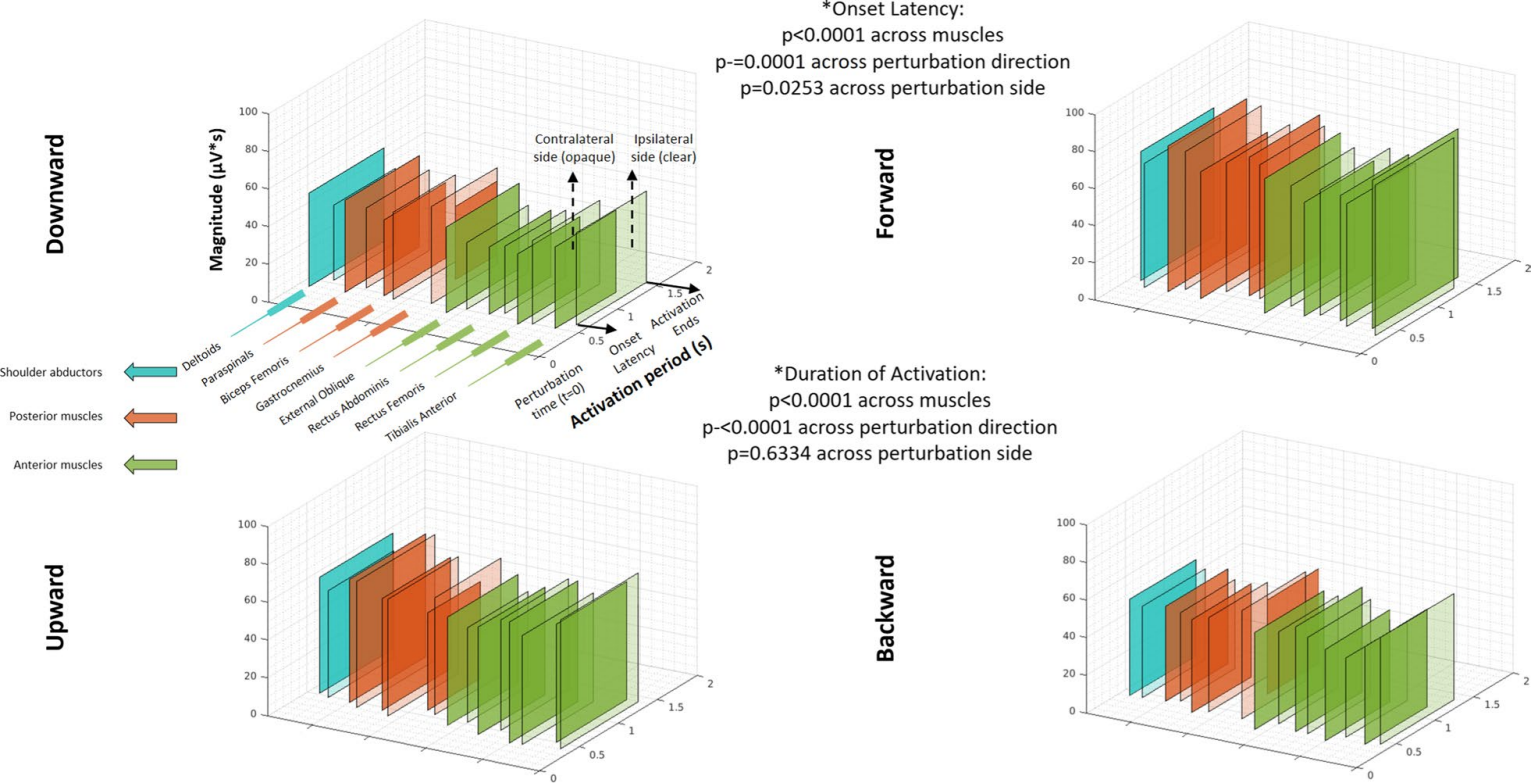
Patterns of muscle activation following perturbations while walking. Description of muscle activation patterns arranged by 3-D rectangles, representing groups of shoulder abductors (blue), posterior muscles (orange) and anterior muscles (green). Clear and opaque rectangles indicate, respectively, muscles in the ipsilateral or contralateral side of perturbation (i.e., side of early foot contact during perturbation). Average values shown from all participants for onset latency and duration of activation (“Activation period” axis), and for activation magnitude (vertical axis). (Additional file 4: Table S2) details the values of EMG parameters during walking conditions. *: p-values observed within the main factors characterizing the ANOVA statistical analysis (see text and Additional file 6 for more details)

Figure 5 illustrates the sequence and magnitude of muscle activations (color-coded) in response to physical perturbations during walking. The figure comprises data on EMG onset latency, duration, and magnitude of each muscle activation seen during walking. Complementing this figure is Additional file 4: Table S2, which details the corresponding numeric values. In addition, Additional file 6 provides the statistical output from the analysis of onset latency and duration of activation during walking conditions.

#### Description of muscle onset patterns of vertical perturbations

Following downward perturbations, the contralateral deltoid had an earlier response (i.e. *onset latency*) in comparison to the ipsilateral deltoid (0.38 ± 0.33s vs. 0.55 ± 0.36s, p = 0.0394), and the ipsilateral gastrocnemius had an earlier response in comparison to the respective contralateral muscle (0.50 ± 0.32s vs. 0.95 ± 0.52s, p < 0.0001). In addition, contralateral external oblique, paraspinal and deltoid muscles were the first to be activated; all of which had a latency significantly shorter than the ipsilateral tibialis anterior and rectus abdominis, and contralateral gastrocnemius (e.g. the contralateral deltoid compared, respectively, to the latter three muscles: p = 0.0036, p = 0.0164 and p < 0.0001).

Following upward perturbations, no differences between ipsilateral and contralateral muscles were found. Both ipsilateral and contralateral rectus femoris were first to respond (e.g. contralateral side: 0.34 ± 0.16s), with a latency significantly shorter than that of the gastrocnemius bilaterally (e.g. contralateral side: 0.60 ± 0.31s; p = 0.0011), the ipsilateral external oblique (p = 0.0184) and contralateral deltoid (p = 0.0228). After the activation of rectus femoris, the ipsilateral biceps femoris and the contralateral rectus abdominis activated, which exhibited earlier onset latencies than the gastrocnemius (p = 0.0074 and p = 0.0050, respectively).

Downward perturbations generated a shorter duration of activation in the contralateral gastrocnemius (0.53 ± 0.29 s) compared to all other muscles; for example, in the comparisons with contralateral tibialis anterior (p = 0.0032) and biceps femoris (p = 0.0020), and with ipsilateral paraspinal (p = 0.0012) and deltoid (p = 0.0027) muscles. In addition, the ipsilateral deltoid had a shorter activation when compared to the contralateral deltoid (0.79 ± 0.29 s vs. 0.95 ± 0.31s, p = 0.0291). The muscles with the longest durations of activation were the contralateral paraspinal, external oblique and deltoid.

After upward perturbations, the contralateral gastrocnemius had the shortest duration of activation (0.67 ± 0.25s) when compared to all other muscles (e.g., when compared to ipsilateral and contralateral rectus abdominis: p = 0.0108 and p = 0.0186 respectively). The only exception to this pattern was with ipsilateral external oblique: 0.77 ± 0.28s, p = 0.1770. The longest durations were evident at the ipsilateral tibialis anterior, contralateral deltoid, and mainly, in both paraspinals; all of these muscles’ durations of activation were significantly longer than those of the contralateral gastrocnemius and ipsilateral external oblique. For example, p < 0.0001 in the comparison between ipsilateral tibialis anterior and contralateral gastrocnemius, and p = 0.0027 when comparing ipsilateral external oblique and paraspinal muscles.

#### Muscle activation—comparison of vertical and horizontal perturbations

Onset latency after downward perturbations was significantly longer (i.e. delayed) when compared to the upward (p = 0.0021), forward (p = 0.0002) and backward (p < 0.0001) perturbations.

Duration of activation after downward perturbations was shorter in comparison to both upward (p = 0.0023) and forward perturbations (p < 0.0001), but not to backward perturbations (p = 0.3786).

### Effects of visual-only perturbations (Figs. 6, 7)

**Fig. 6:**
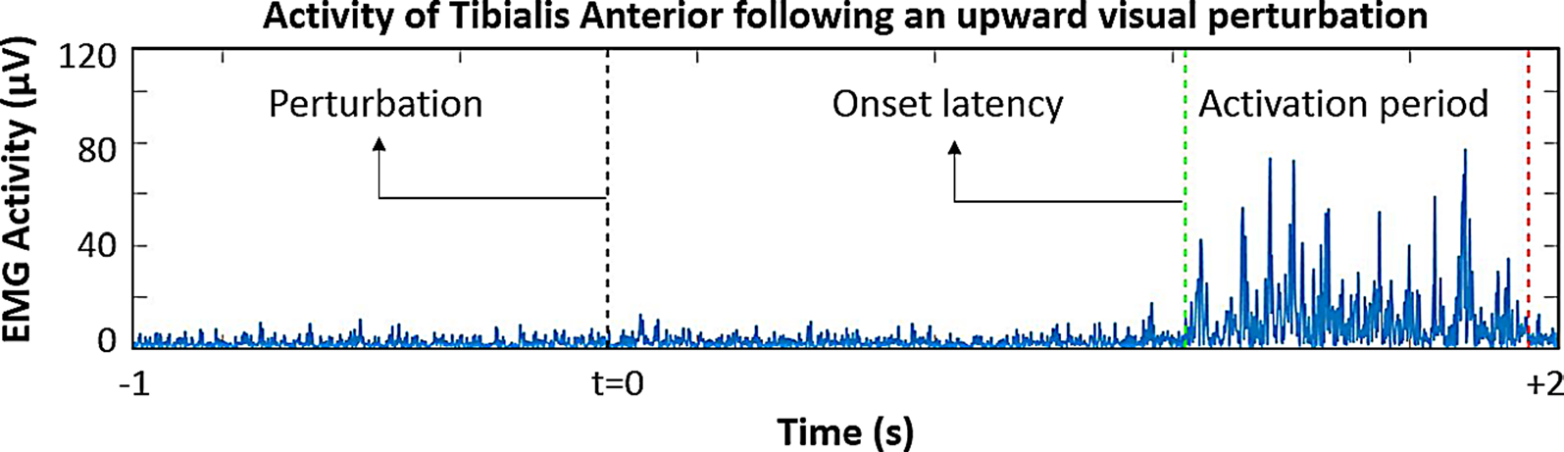
Electromyography (EMG) activity driven by visual perturbations. Filtered and rectified EMG activity of left tibialis anterior following an upward visual perturbation while standing. The figure shows a time window from one second before to two seconds after visual perturbation. Onset latency was 1.2 s, duration of activation was 723 ms, and magnitude of activation was 3.85 μV*s

**Fig. 7:**
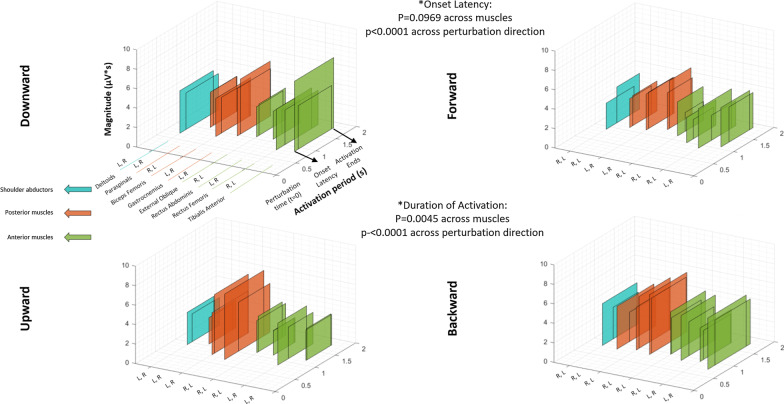
Patterns of muscle activation following visual perturbations. Description of muscle activation patterns arranged by 3-D rectangles representing groups of shoulder abductors (blue), posterior muscles (orange) and anterior muscles (green). Figures show average values (from all participants) of onset latency and duration of activation (“Activation period” axis), and activation magnitude (vertical axis). (Additional file 5: Table S3) details the values of EMG parameters triggered by visual perturbations. The overlapping rectangles depict left (L) and right (R) body sides. The letters indicate the shorter and longer onset latency, respectively. *: p-values observed within the factors characterizing the ANOVA statistical analysis (see text and Additional file 6 for more details)

Effective muscle activation (i.e., removing the “no response” cases, see Methods) following visual perturbations was evident 118 to 143 times per participant out of 192 exposures to visual perturbations (exposures being defined by 3 repetitions of 4 perturbation directions across 16 muscles), for an average of 67.7% effective muscle responses. For an example of a response to a visual perturbation, see Fig. 6.

Figure 7 illustrates the sequence and magnitude of muscle activations (color-coded) in response to visual perturbations. Complementing this figure is Additional file 5: Table S3, which details the numeric values. In addition, Additional file 6 provides the statistical output from the analysis of onset latency and duration of activation during visual conditions.

Forward visual perturbations led to the most delayed onset latencies, significantly longer when compared to the downward (p = 0.0002), upward (p = 0.0015) and backward (p < 0.0001) visual perturbations. No statistical effect on onset latency was found across muscles (p = 0.0969) (Fig. 7).

Longest duration of activation was observed in tibialis anterior, gastrocnemius, and biceps femoris. In contrast, the left rectus abdominis presented with the shortest duration of activation (e.g., compared with the left side of the aforementioned three muscles, respectively, p = 0.0004, p = 0.0011 and p = 0.0014). Backward visual perturbations led to the longest durations of activation, whereas forward visual perturbations led to the shortest durations of activation. For instance, duration of activation following forward visual perturbations was shorter than downward (p = 0.0014) and backward (p ≤ 0.0001) visual perturbations, but not when compared to upward visual perturbations (p=0.0851).

Within visual perturbations, different perturbation directions did not lead to significantly different magnitudes within each muscle (e.g., p = 0.5858 in the analysis for right biceps femoris).

## Discussion

### Summary of results and study strengths

**Fig. 8:**
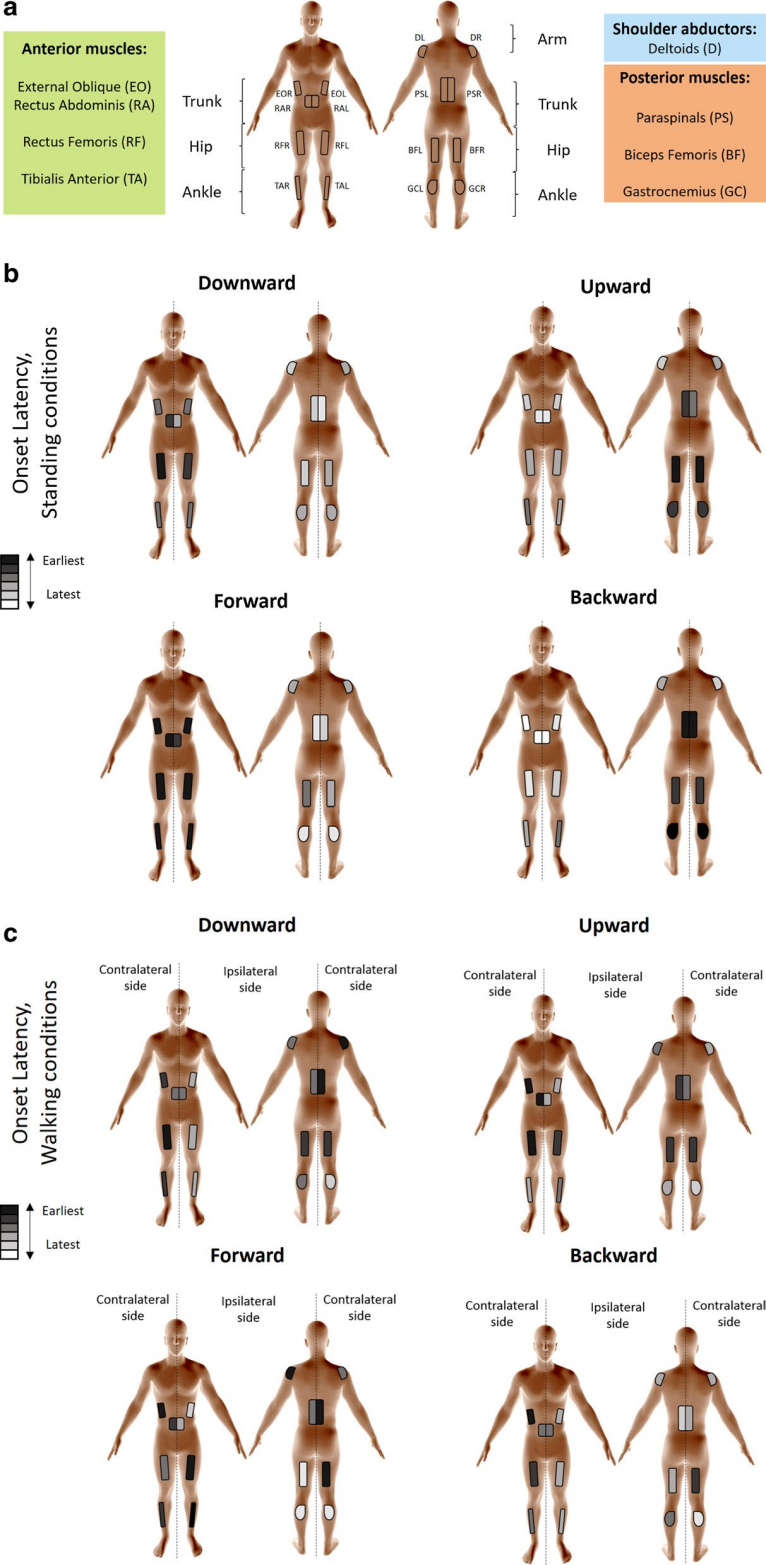
Schematic representation of muscle activation patterns. **a** Assessed muscles grouped by anterior muscles (TA: tibialis anterior; RF: rectus femoris; RA: rectus abdominis; and EO: external oblique), posterior muscles (GC: gastrocnemius lateralis; BF: biceps femoris; and PS: paraspinals) and shoulder abductors (D: deltoid medial). L or R at the end of abbreviation refer to left/right side (e.g. TAL: tibialis anterior left). The figures show patterns of electromyography activation arranged from earlier to later onset latencies within horizontal and vertical perturbations following **b** standing perturbations (combining all three sensory conditions) and **c** walking perturbations (combining both left and right perturbations). A color-code identifies those muscles responding earlier (darker) or later (whiter) in each perturbation direction

We show muscle activation patterns of leg, trunk, and shoulder muscles that characterize balance-correcting responses following vertical perturbations (as compared to horizontal perturbations) during standing and walking, and in response to visual perturbations. We hypothesized that a coordinated activation of inter-segmental muscles would define postural control responses and that muscle response patterns would differ among vertical versus horizontal perturbations. The results largely support these hypotheses (Additional file 1 details how the results correspond to a priori predictions). Indeed, muscle activation patterns were consistent with counteracting the induced postural perturbation in a direction-specific manner, such that forward perturbations induced a backward fall counteracted primarily by distal anterior muscle activation; backward perturbations induced a forward fall counteracted primarily by distal posterior muscle activation; upward perturbations induced body compression counteracted primarily by proximal posterior activation; and, downward perturbations induced vertical body extension counteracted primarily by proximal anterior flexor activation. Specifically, vertical perturbations usually led to early activation of rectus and biceps femoris, and to otherwise later onset latencies, shorter durations of activation, and lower activation magnitudes in comparison to horizontal perturbations, which in contrast triggered an initial activation of tibialis anterior and gastrocnemius muscles (Fig. 8).

Visual conditions modulated muscle activation patterns. Eyes-closed conditions led to longer durations of activation and larger activation magnitudes, whereas (sensory incongruent) static-camera conditions led to longer onset latencies. We show that mere visual perturbations can elicit muscle activation, although with a level of activation magnitude significantly lower when compared to physical perturbations.

Our results suggest that vertical perturbations promote differentiated balance-correction strategies oriented to prioritize trunk and hip configuration, and that the availability and manipulation of visual cues through VR can modulate inter-segmental muscle activation.

Our study presents with some strengths. In particular, we introduce a comprehensive, full-body analysis of balance-correcting muscle responses following vertical perturbations. It enriches the literature because research had historically favoured horizontal perturbations and lower-limb muscles. In addition, the integrated, large-scale VR facility used for this study provides basis for further application in the growing adoption of digital tools of human-computer interaction, including the incorporation of VR for healthcare applications and clinical therapy.

### Comparison with the literature

Our observation that downward perturbations led to a predominantly initial activation of proximal muscles is consistent with findings showing that unexpected falls lead to landing-like muscular control [22]. This is in accordance with other studies suggesting that a flexed trunk reduces ground reaction forces while landing [45] and that an erect landing pattern represents injury risk [46]. The quasi-opposite patterns of muscle activation triggered by upward perturbations are compatible with another study [12], which showed that upward (compared to downward) perturbations lead to opposed changes in body height (e.g. flexion of ankles and knees) and vertical loading. Our study builds on the latter study, particularly, by including an assessment of trunk and shoulder muscles in addition to the leg muscles.

Furthermore, our results in respect to horizontal perturbations are largely consistent with previous results, showing a dorsal-ventral pattern; i.e. a main activation of tibialis anterior, rectus femoris, and rectus abdominis ensuing anterior, forward surface translation, and opposing activation of gastrocnemius, biceps femoris, and paraspinals after posterior, backward translations [5–7, 15–17, 21]. Our findings that rectus abdominis, external oblique, and paraspinals had major balance-correcting roles support their function as prime movers for trunk stabilization during postural control tasks ensuing unexpected perturbations [6]. Our study additionally contributes, when compared to the aforementioned papers, by including vertical perturbations in the analysis of muscle activation that characterize postural readjustment.

In addition, the quasi-opposite functional characteristics of initially-activated muscle groups responding to upward and downward perturbations are comparable to a previous study reporting that groups of muscles responding to unexpected lowered and level surfaces during walking were almost the exact opposite to each other [25]. The findings of their study show these quasi-opposite patterns based on the EMG analysis of four leg muscles, whereas our results expand their observations by describing similar findings from trunk muscles such as rectus abdominis and paraspinals, and by including standing conditions. These additions provide more context into the direction-specific muscle activation patterns needed to render a functional counteractive response to the induced postural perturbation. Moreover, our finding of a leading contralateral deltoid activation following downward perturbations while walking seems consistent with the whole-body coordinated reaction theory [29] and supports the essential role of arm motion for perturbation recovery when the trunk moves downward [47, 48].

The abrupt abdominal depression caused by downward perturbations [30], combined with the characteristic locomotor body propulsion [49], might explain the distinct early activation of trunk extensors that followed downward perturbations during walking conditions (in comparison to the elicited early activation of trunk flexors in standing conditions). Presumably, while a trunk flexion (i.e. rectus abdominis, external oblique activation) is expected to counter body elongation following downward perturbations while standing, body inclination and locomotor propulsion are additional biomechanical constraints associated with walking, during which the activation of trunk extensors (i.e. paraspinals) is expected to counter the body shortening induced by body inclination/propulsion. Previous studies reporting trunk extension while walking on camouflaged drops [50] and paraspinals activation elucidated by unexpected foot-in-hole scenarios [26] support our findings. Although the first study [50] focused on kinetic, kinematic parameters analysis and the second [26] mainly investigated the effect of prior knowledge on fast muscle responses, we hypothesize that similar postural strategies explain the distinctive early activation of (contralateral) trunk extensors/flexors during standing/walking conditions. In agreement with our results, a previous study found that the pattern of EMG activity during vertical, locomotor-like perturbations contrasted with the EMG pattern following vertical displacements during standing [12].

The relatively long onset latency after vertical perturbations (> 600 ms) while standing might be due to the perturbation intensity [51, 52]. While in walking conditions, perturbations were characterized by a 20cm displacement within 300 ms (~0.45 g), platform displacements during standing conditions were of 12 cm within 1000 ms; i.e., <0.2 g. In comparison, free-fall studies found activations occurring 74.2 ms after fall initiation with a 160 ms fall duration [22]. EMG activity depends on the height and duration of the fall [22, 41, 53, 54]. Thus, generalizability within vertical perturbations may be limited across studies of different perturbation magnitudes.

Previous studies show that 80% of participants experience vection (i.e. self-motion sensation) when exposed to moving visual scenes [55]. We observed a muscle response rate of 68% after visual perturbations. Our observed onset latencies ensuing visual perturbations are in agreement with the onset range of 0.5–2 s characterizing postural readjustments following motion of visual scenes previously reported in studies investigating vection [55, 56].

Our observation that responses were evident merely from changes in the visual scenes and without any physical change in the standing surface, in combination with the lack of efficiency of responses evident when vision was unavailable, indicate that an initial experience of vection (or environmental transition) is sufficient to induce postural responses and supports the notion that visual inputs contribute to an efficient response.

In addition, our findings are consistent with the suggestion that several people may be insensitive to visual perturbations [57]. We observed that onset latencies following visual perturbations were generally delayed in comparison to onset latencies after physical perturbations. At least three stages may explain longer onset latencies after visual perturbations: eliciting vection, realizing the conflict, and adjusting posture [36, 57]. Nashner and Berthoz [36], suggest that incongruent visual inputs lead to a sensory reweighting process that requires adaptation of the motor system, and that rapid and slow visual influences upon balance have different functions. While they agree with other authors [55] that delayed responses work as slow compensatory input to posture, they further suggest that attenuation of initial activity may serve to “withhold potentially erroneous responses until an unexpected discongruence” among sensory inputs can be resolved. Thus, delayed responses and variability to elicit responses to visual perturbations may reflect the neural processing delays of the visual modality, differences in weighting of the visual modality to define postural responses, as well as the ability and extended time necessary to resolve incongruent feedback.

### Limitations, future directions and implications

There are some limitations related to our study. The experimental paradigm required a definition of perturbation intensities. We introduced *slow* perturbation intensities to avoid stepping strategies (we also encouraged participants not to step during the standing experimental conditions). Nevertheless, as noted above, the muscle activation patterns observed in our experiments are similar to the ones previously reported in the literature, in particular following backwards and forward perturbations [6, 16, 17], which suggests that perturbation intensity in our study was sufficiently fast to induce the previously reported postural corrections.

Our inability to completely mitigate expectation of perturbations represents another methodological limitation in our study. We attempted to reduce the potential for pre-planned response priming by randomizing the direction of the perturbation and by introducing a random epoch between trials. Although these protocols likely mitigate pre-planning and priming of direction-specific responses or optimizing the timing of the response, the participants were aware of an impending postural perturbation. A different experimental design would be required to elicit postural responses from truly unexpected perturbations. Nonetheless, although the environmental validity of our protocol to represent real-world unexpected losses of balance may be reduced, the experimental design enabled a controlled environment to test the primary hypotheses pertaining to the sensory-motor control of direction-specific postural response patterns.

Another limitation relates to the participant sampling and VR laboratory conditions. Our experiments exposed young, healthy participants to VR paradigms within a room-sized computer-assisted rehabilitation environment. These characteristics limit the extent to which our results may generalize to a broader population. Future research can investigate the feasibility of translating the described experimental paradigms for persons with sensory, motor and cognitive conditions in real-world scenarios.

A potential limitation was the method used for determining EMG onset, which within a three-second window normalizes the signal and determines the largest deviation from the integrated EMG. However, after visual comparison on our experimental data, this method was found to be more robust and to lead to fewer false onsets when compared to the conventional methods that determine EMG onset based on threshold (mean plus X standard deviations) [58]. In addition, our calculation of EMG magnitude based on numerical integration throughout the activation period presents with some strengths and weaknesses. For instance, other methods such as peak EMG would allow evaluating the latency of the maximum response, but without evaluating bursts of muscle activity [52]. The strength of numerical integration, however, relies on the ability to provide an average EMG activation magnitude that can be comparable across different types and conditions of perturbations.

Our findings may have translational benefits for balance and gait rehabilitation, such as for people at risk of falling, and for implementing perturbation-based treatments [9, 11]. Clinicians may incorporate the reported paradigm of unexpected perturbations within reactive postural control training programs. Rehabilitation treatments may initially focus on the response-leading muscles. For instance, the implementation of downward-perturbation training for activating deltoid muscles and increasing shoulder abduction might modulate the coordination of arm movements with trunk and leg motion to improve balance reactions during locomotion. In addition, gait treatments may expose patients to upward and downward perturbations for rehabilitation of trunk flexors and extensors, respectively. Else, standing upward-perturbation training may be used for stimulating hamstring activation. Given our observations that groups of muscles respond in unique sequence to different directions of perturbation, balance treatments can incorporate treatment approaches focused on specific groups of muscles, according to specific therapeutic goals. The differences in amplitude of activation across directions may also lend to progressive therapies in order to address a broader range of impairments across clinical populations while also enabling training of more diverse responses to more diverse perturbation characteristics. Furthermore, with the growing interest in the utilization of exoskeleton, robotic devices designed to enable locomotion in, for example, persons suffering from paraplegia [59, 60], it is crucial to program these robots to respond to any unexpected physical perturbation in a manner ensuring user safety. Knowledge on natural muscle activation patterns in humans is imperative for such programming, as well as for programming robotic devices used for gait rehabilitation [8, 10].

Notwithstanding, caution should be taken when translating the findings of our study, such as when planning to use our experimental paradigm for investigating falls in older adults or persons with neurological conditions, or when using our findings for the design and planning of perturbation-based balance and gait rehabilitation programs. Our study provides a mechanistic understanding of muscle activation patterns following perturbations in different directions that include vertical perturbations, but there are vulnerable populations that can present with different postural strategies and balance behaviors (e.g. elderly, post-stroke patients), thereby requiring further study on these populations of interest.

Future directions from the present study relate to investigating the role of visual pathways in generating balance-correcting muscular responses. We observed that magnitude of muscle activation, for instance, was progressive: smallest after visual perturbations, followed by (physical perturbations) dynamic-camera, static-camera, and finally, eyes-closed conditions that usually led to largest magnitudes. Visual sensory cues have been suggested to be suitable for balance-control regulation [61, 62], and for locomotion modulation related to surface inclination changes [35]. Understanding the multisensory integration determining muscle activation patterns for postural control can have translational benefits for patients with sensory-integration dysfunctions, either to entrain compensations or to identify sources of sensory impairment.

This study also provides insights on how mere visual perturbations and the manipulation of visual cues during standing following physical perturbations may activate muscle response patterns and modulate postural control strategies. Such information can help for optimizing the design and planning of rehabilitation strategies using immersive VR tools in a safe and controlled environment. In terms of training intensity, for instance, we observed that horizontal physical perturbations triggered the most intense muscle responses, whereas visual perturbations elicited the lowest magnitudes of activation. Physical vertical perturbations, therefore, represent a medium-intense set of perturbations that will not necessarily be destabilizing, and can be incorporated during early stages of balance-reaction therapies and in research projects on falls risk. Our paradigm of visual perturbations can also be incorporated to determine levels of responsiveness towards VR visual stimuli.

## Conclusion

We conclude that a coordinated activation of muscles across the body characterizes balance-correcting responses to vertical perturbations during standing and gait that are specific and appropriate to the direction of induced displacement. Major trunk muscles such as paraspinals, rectus abdominis, and external oblique were among the first to activate during postural responses to vertical perturbations, which contrast the initial activation of distal leg muscles in response to horizontal perturbations. Further, the availability of visual cues supports more efficient responses, whereas visual conflict can affect the timely triggering of balance corrective responses.

## Supplementary Information

**Additional file 1:** Rationale and description of specific predictions/hypotheses about muscle activations.

**Additional file 2: Video S1.**

**Additional file 3: Table S1.** Values of EMG parameters during standing conditions.

**Additional file 4: Table S2.** Values of EMG parameters during walking conditions.

**Additional file 5: Table S3.** Values of EMG parameters after visual perturbations.

**Additional file 6:** Entire ANOVA table outputs from the statistical analysis of onset latency and duration of activation in standing and walking conditions.

## Data Availability

The datasets used and/or analyzed during the current study are available from the corresponding author on reasonable request.

## Abbreviations

BF: Biceps femoris
BMI: Body Mass Index
COM_x_: Extrapolated center of mass
D: Deltoid medial
EMG: Electromyography
EO: External oblique
GC: Gastrocnemius lateralis
PS: Paraspinal
RA: Rectus abdominis
RF: Rectus femoris
SSV: Steady-state velocity
TA: Tibialis anterior
VR: Virtual Reality
