# Supplementary material for: Patterns of whole-body muscle activations following vertical perturbations during standing and walking
Source: J Neuroeng Rehabil. 2021 May 6;18:75. doi: 10.1186/s12984-021-00836-0 (PMC8101216; doi:10.1186/s12984-021-00836-0)
Supplement: Supplementary file 1 — Additional file 1: Rationale and description of specific predictions/hypotheses about muscle activations. [file 12984_2021_836_MOESM1_ESM.docx]

**SUPPLEMENTARY FILE #1**

***Rationale and description of specific a priori predictions/hypotheses about muscle activations***

Because vertical perturbations lead to different body movements than horizontal perturbations (implying different balance-correcting COM movements), we hypothesized that muscle activation patterns in response to vertical perturbations will differ from those in response to horizontal perturbations.

Several predictions emerge from this hypothesis in *standing* conditions:

1) We predict a major, initial function of spinal flexors/extensors in maintaining balance after vertical perturbations, given the primary role of the trunk as stabilizer/prime-mover after perturbations in multiple directions [[1-6](#_ENREF_1)].

2) Because the nervous system employs antagonistic co-activation (stiffening strategy) during circumstances of task uncertainty and postural threat [[7-11](#_ENREF_7)], presumably for delaying compensatory responses to allow sufficient time for weighting available sensory inputs [[4](#_ENREF_4), [12](#_ENREF_12)], we predict a co-activation of upper and lower leg agonist/antagonists due to the unpredictable nature of the perturbations employed in this study.

3) Because actual perturbing events mostly occur during walking [[13](#_ENREF_13), [14](#_ENREF_14)], our study further aimed to address predictions regarding muscle activations in response to vertical perturbations during human locomotion. Concerning the *walking* conditions our prediction mirrors prediction 1, in which we anticipate a primary role of the trunk [[5](#_ENREF_5), [15](#_ENREF_15)]. We, however, do anticipate the context of walking will elicit unique postural response strategies to vertical perturbations.

*The role of vision*

Moreover, we aimed to test whether postural response strategies to vertical perturbations depend on current sensory feedback as they do for horizontal perturbations [[1](#_ENREF_1), [16](#_ENREF_16), [17](#_ENREF_17)], and to investigate whether an internal model comparing expected versus actual sensory feedback, influences responses to vertical perturbations [[6](#_ENREF_6), [18](#_ENREF_18)]. Therefore, our experimental paradigm while standing included physical perturbations with three sensory feedback conditions (eyes closed, static visual scene, and dynamic visual scene during surface perturbations), as well as visual perturbations (i.e.; only visual scenes move). Here, we focused on the modulation of balance-correcting responses caused by the manipulation of visual cues. Our focus on visual cues is due to the critical role of vision in maintaining equilibrium [[19-21](#_ENREF_19)], and because visual perturbations simulate gravitational changes [[22](#_ENREF_22), [23](#_ENREF_23)] and trigger muscle activation in different paradigms [[20](#_ENREF_20), [24](#_ENREF_24), [25](#_ENREF_25)].

***How the results correspond to a priori predictions and hypotheses***

In keeping with prediction 1, which hypothesized a major, initial function of spinal flexors/extensors in maintaining balance after vertical perturbations, we observed that such trunk flexors as rectus abdominis and external oblique were among the initially activated muscles following downward perturbations during standing, along with rectus femoris and tibialis anterior, all of them anterior muscles. Antagonists trunk extensors such as paraspinals, meanwhile, were the last responding muscles (Figure 3). In contrast, following upward perturbations, paraspinals were among the first-responding muscles, usually followed by biceps femoris and gastrocnemius. Antagonist flexors such as external oblique and rectus abdominis, in turn, were among the last muscles to be activated. These results suggest that a coordinated trunk/hip flexion and extension characterize postural strategies following downward and upward perturbations during standing, respectively.

In regards to prediction 2, which expected a co-activation of upper and lower leg agonist/antagonists due to the unpredictable nature of the perturbations employed in this study, we indeed observed a co-activation of gastrocnemius and tibialis anterior following vertical perturbations; however, contrary to our expectations, upper leg antagonists (biceps and rectus femoris) systematically activated at significantly different onset latencies. Downward perturbations led to a fastest activation of rectus femoris in comparison to biceps femoris, whereas upward perturbations led to opposite patterns. While the rectus femoris flexes the hip and extends the knee, the biceps femoris extends the hip and flexes the knee. Because downward perturbations systematically led to a fastest activation of anterior muscles (in contrast with upward perturbation that promptly activated posterior muscles), our results suggest that the role of upper leg muscles is mainly trunk stabilization. This observation appeared more evident during static-camera conditions (Figure 3). Furthermore, we found intriguing similarities concerning the coordinated activation of trunk and leg muscles between downward and forward perturbations, and between upward and backward perturbations. Whereas fastest responding muscles to forward perturbations comprised tibialis anterior, rectus femoris, rectus abdominis and external oblique (as occurred after downward perturbations), respective antagonists gastrocnemius, biceps femoris and paraspinals defined initial responses to backward (as occurred after upward) perturbations.

Our results in walking conditions support prediction 3, where we anticipated that the context of walking will elicit unique postural response strategies to vertical perturbations, still showing a major trunk response mirroring prediction 1, namely that trunk/hip action-driving muscles would have a predominantly faster activation. For example, contralateral external oblique and paraspinals (along with the contralateral deltoid) were the first muscles to be activated after downward perturbations; while rectus femoris was the most promptly activated muscle after upward perturbations.

Walking is more efficient when the arm swings upward while the trunk moves downward, and when the shoulder is in maximum extension at the time of ipsilateral foot contact [[26](#_ENREF_26)]. Additional studies suggest a coupling of arm-trunk muscle activation patterns [[27](#_ENREF_27), [28](#_ENREF_28)]. It is therefore expected that balance-correcting responses to vertical perturbations during walking will be characterized by a coordinated activation of trunk and shoulder muscles; and an asymmetric bilateral muscle activation pattern of leg, trunk and shoulder; i.e. contralateral muscles will have activation patterns opposite to ipsilateral muscles. Studies on interlimb coordination during stance [[29](#_ENREF_29)] and walking [[6](#_ENREF_6), [10](#_ENREF_10), [18](#_ENREF_18), [30](#_ENREF_30)] support the latter prediction. Indeed, we observed a significant early activation of the contralateral deltoid and a dominant initial activation of contralateral muscles following downward perturbations, which suggest an asymmetric (from contralateral to ipsilateral) strategy to maintain balance in response to unexpected perturbations.

We found that visual perturbations, including those with vertical displacement of visual scenes, can activate inter-segmental muscle response patterns. Muscle activation in forward visual perturbations usually presented with longer onset latencies and shorter durations of activation when compared to other visual perturbation directions (Figure 7). Reasonably, it might be because human vision usually focuses on objects/environments that are approaching (or being approached) thus modulating reactions to nearing visual scenes (i.e. forward) [[31](#_ENREF_31)].

Our findings suggest an effect of visual cues during balance-correcting responses while standing. We hypothesized that conditions that induce incongruent sensory input would lead to more challenged balance-correcting responses characterized by longer latency, duration and increased magnitude of muscle activation. We observed that eyes-closed conditions led to the largest activation magnitudes and to longer durations of muscle activation. In addition, activation magnitudes in both eyes-closed and static-camera conditions were larger than in dynamic-camera conditions. In sum, conditions with sensory conflict or absence of visual cues resulted in more challenging postural tasks, thus, eliciting increased muscular responses [[1](#_ENREF_1), [4](#_ENREF_4), [17](#_ENREF_17)].

**References**

1. Chvatal SA, Ting LH: **Common muscle synergies for balance and walking**. *Frontiers in computational neuroscience* 2013, **7**:48.

2. Do M, Breniere Y, Bouisset S: **Compensatory reactions in forward fall: are they initiated by stretch receptors?** *Electroencephalography and clinical Neurophysiology* 1988, **69**(5):448-452.

3. Henry SM, Fung J, Horak FB: **EMG responses to maintain stance during multidirectional surface translations**. *Journal of Neurophysiology* 1998, **80**(4):1939-1950.

4. Horak F, Shupert C, Dietz V, Horstmann G: **Vestibular and somatosensory contributions to responses to head and body displacements in stance**. *Experimental Brain Research* 1994, **100**(1):93-106.

5. Mackinnon CD, Winter DA: **Control of whole body balance in the frontal plane during human walking**. *Journal of biomechanics* 1993, **26**(6):633-644.

6. Shinya M, Fujii S, Oda S: **Corrective postural responses evoked by completely unexpected loss of ground support during human walking**. *Gait & posture* 2009, **29**(3):483-487.

7. De Luca CJ, Mambrito B: **Voluntary control of motor units in human antagonist muscles: coactivation and reciprocal activation**. *Journal of neurophysiology* 1987, **58**(3):525-542.

8. Doorenbosch CA, Harlaar J, van Ingen Schenau GJ: **Stiffness control for lower leg muscles in directing external forces**. *Neuroscience letters* 1995, **202**(1-2):61-64.

9. Misiaszek JE, Krauss EM: **Restricting arm use enhances compensatory reactions of leg muscles during walking**. *Experimental brain research* 2005, **161**(4):474-485.

10. Nazifi MM, Yoon HU, Beschorner K, Hur P: **Shared and task-specific muscle synergies during normal walking and slipping**. *Frontiers in human neuroscience* 2017, **11**:40.

11. Santello M, McDonagh MJ: **The control of timing and amplitude of EMG activity in landing movements in humans**. *Experimental Physiology* 1998, **83**(6):857-874.

12. Pew RW: **Levels of analysis in motor control**. *Brain Research* 1974, **71**(2-3):393-400.

13. Courtney TK, Sorock GS, Manning DP, Collins JW, Holbein-Jenny MA: **Occupational slip, trip, and fall-related injuries–can the contribution of slipperiness be isolated?** *Ergonomics* 2001, **44**(13):1118-1137.

14. Talbot LA, Musiol RJ, Witham EK, Metter EJ: **Falls in young, middle-aged and older community dwelling adults: perceived cause, environmental factors and injury**. *BMC public health* 2005, **5**(1):86.

15. Müller R, Tschiesche K, Blickhan R: **Kinetic and kinematic adjustments during perturbed walking across visible and camouflaged drops in ground level**. *Journal of biomechanics* 2014, **47**(10):2286-2291.

16. Hammond P, Merton P, Sutton GG: **Nervous gradation of muscular contraction**. *British medical bulletin* 1956, **12**(3):214-218.

17. Horak FB, Nashner LM: **Central programming of postural movements: adaptation to altered support-surface configurations**. *Journal of neurophysiology* 1986, **55**(6):1369-1381.

18. van der Linden MH, Marigold DS, Gabreëls FJ, Duysens J: **Muscle reflexes and synergies triggered by an unexpected support surface height during walking**. *Journal of neurophysiology* 2007, **97**(5):3639-3650.

19. Edwards A: **Body sway and vision**. *Journal of experimental psychology* 1946, **36**(6):526.

20. Santello M, McDonagh MJ, Challis JH: **Visual and non‐visual control of landing movements in humans**. *The Journal of physiology* 2001, **537**(1):313-327.

21. Travis RC: **An experimental analysis of dynamic and static equilibrium**. *Journal of Experimental Psychology* 1945, **35**(3):216.

22. Dichgans J, Held R, Young LR, Brandt T: **Moving visual scenes influence the apparent direction of gravity**. *Science* 1972, **178**(4066):1217-1219.

23. Cano Porras D, Zeilig G, Doniger GM, Bahat Y, Inzelberg R, Plotnik M: **Seeing gravity: gait adaptations to visual and physical inclines–A virtual reality study**. *Frontiers in Neuroscience* 2020, **13**:1308.

24. Berthoz A, Pavard B, Young L: **Perception of linear horizontal self-motion induced by peripheral vision (linearvection) basic characteristics and visual-vestibular interactions**. *Experimental brain research* 1975, **23**(5):471-489.

25. Lestienne F, Soechting J, Berthoz A: **Postural readjustments induced by linear motion of visual scenes**. *Experimental brain research* 1977, **28**(3-4):363-384.

26. Murray MP, Sepic S, Barnard E: **Patterns of sagittal rotation of the upper limbs in walking**. *Physical therapy* 1967, **47**(4):272-284.

27. Bruijn SM, Meijer OG, Beek PJ, van Dieën JH: **The effects of arm swing on human gait stability**. *Journal of experimental biology* 2010, **213**(23):3945-3952.

28. Nashner LM, Forssberg H: **Phase-dependent organization of postural adjustments associated with arm movements while walking**. *Journal of Neurophysiology* 1986, **55**(6):1382-1394.

29. Dietz V, Horstmann G, Berger W: **Interlimb coordination of leg-muscle activation during perturbation of stance in humans**. *Journal of neurophysiology* 1989, **62**(3):680-693.

30. Bachmann V, Müller R, Van Hedel H, Dietz V: **Vertical perturbations of human gait: organisation and adaptation of leg muscle responses**. *Experimental brain research* 2008, **186**(1):123-130.

31. Apthorp D, Nagle F, Palmisano S: **Chaos in balance: non-linear measures of postural control predict individual variations in visual illusions of motion**. *PloS one* 2014, **9**(12):e113897.
