# Supplementary material for: Patterns of whole-body muscle activations following vertical perturbations during standing and walking
Source: J Neuroeng Rehabil. 2021 May 6;18:75. doi: 10.1186/s12984-021-00836-0 (PMC8101216; doi:10.1186/s12984-021-00836-0)
Supplement: Supplementary file 3 — Additional file 3: Table S1. Values of EMG parameters during standing conditions. [file 12984_2021_836_MOESM3_ESM.pdf]

**Table 1:** Values of EMG parameters during standing conditions

| | | Deltoid left | | | Deltoid right | | | Paraspinal left | | |
|---|---|---|---|---|---|---|---|---|---|---|
| | | DC | SC | EC | DC | SC | EC | DC | SC | EC |
| DP | OL (s) | 1.08±0.45 | 1.23±0.41 | 0.97±0.44 | 0.75±0.49 | 1.14±0.38 | 0.94±0.39 | 1.07±0.27 | 1.16±0.27 | 1.03±0.11 |
| | DA (s) | 0.69±0.41 | 0.53±0.29 | 0.74±0.41 | 0.91±0.51 | 0.57±0.29 | 0.69±0.22 | 0.68±0.26 | 0.65±0.28 | 0.76±0.22 |
| | MAG (µV*s) | 5.70±4.29 | 4.14±3.63 | 7.75±5.26 | 6.15±3.81 | 4.13±3.37 | 8.77±7.67 | 6.37±4.76 | 5.41±4.37 | 9.71±6.78 |
| UP | OL (s) | 0.84±0.35 | 1.09±0.34 | 0.95±0.40 | 0.78±0.34 | 1.10±0.42 | 0.83±0.41 | 0.68±0.27 | 0.81±0.51 | 0.72±0.37 |
| | DA (s) | 0.84±0.32 | 0.68±0.39 | 0.68±0.22 | 0.86±0.33 | 0.69±0.33 | 0.80±0.34 | 0.67±0.25 | 0.63±0.37 | 0.73±0.32 |
| | MAG (µV*s) | 6.04±6.36 | 3.53±3.11 | 4.84±2.97 | 5.53±3.42 | 3.41±2.50 | 5.91±4.69 | 5.40±3.78 | 3.56±2.30 | 6.45±5.55 |
| FP | OL (s) | 0.54±0.43 | 0.44±0.12 | 0.45±0.17 | 0.49±0.20 | 0.51±0.16 | 0.50±0.14 | 0.63±0.34 | 0.62±0.19 | 0.57±0.19 |
| | DA (s) | 0.86±0.40 | 1.09±0.27 | 1.06±0.24 | 0.81±0.29 | 0.99±0.28 | 1.04±0.27 | 0.76±0.29 | 0.92±0.22 | 0.93±0.19 |
| | MAG (µV*s) | 17.49±12.77 | 29.30±15.98 | 32.73±17.11 | 15.46±10.74 | 26.2±14.78 | 30.79±19.99 | 16.81±9.75 | 27.93±16.79 | 30.82±16.60 |
| BP | OL (s) | 0.54±0.26 | 0.61±0.33 | 0.56±0.31 | 0.60±0.30 | 0.68±0.24 | 0.66±0.19 | 0.37±0.09 | 0.37±0.13 | 0.36±0.11 |
| | DA (s) | 0.83±0.21 | 1.00±0.32 | 1.09±0.37 | 1.09±0.31 | 1.02±0.19 | 0.98±0.26 | 0.99±0.30 | 0.99±0.328 | 1.06±0.25 |
| | MAG (µV*s) | 13.81±7.39 | 15.46±8.61 | 19.89±11.88 | 15.24±7.26 | 13.86±5.15 | 16.53±8.25 | 16.66±7.33 | 17.07±6.73 | 21.66±10.97 |

**Legend**. DP, UP, FP and BP represent, respectively, downward, upward, forward and backward perturbations. OL: onset latency; DA: duration of activation; MAG: activation magnitude; DC: dynamic camera; SC: static camera; EC: eyes closed.
Color codes: blue represent shoulder abductors, orange posterior muscles and green anterior muscles.

**Table 1:** Values of EMG parameters during standing conditions

| | | DC | SC | EC | DC | SC | EC | DC | SC | EC |
|---|---|---|---|---|---|---|---|---|---|---|
| **DP** | **OL (s)** | 1.20±0.30 | 1.20±0.26 | 1.03±0.22 | 1.07±0.24 | 1.06±0.29 | 1.05±0.18 | 0.96±0.24 | 1.08±0.39 | 0.88±0.25 |
| | **DA (s)** | 0.54±0.29 | 0.47±0.25 | 0.76±0.18 | 0.70±0.22 | 0.71±0.25 | 0.81±0.20 | 0.88±0.21 | 0.67±0.29 | 0.95±0.28 |
| | **MAG (µV*s)** | 5.44±4.75 | 4.38±3.88 | 9.57±6.72 | 6.24±3.40 | 5.97±4.46 | 10.49±7.04 | 8.19±4.44 | 5.90±3.47 | 11.49±7.71 |
| **UP** | **OL (s)** | 0.68±0.49 | 0.86±0.55 | 1.02±0.53 | 0.68±0.41 | 0.50±0.39 | 0.74±0.50 | 0.53±0.26 | 0.78±0.45 | 0.69±0.45 |
| | **DA (s)** | 0.90±0.43 | 0.66±0.40 | 0.67±0.46 | 0.77±0.31 | 1.05±0.41 | 0.83±0.37 | 1.01±0.37 | 0.85±0.38 | 0.93±0.39 |
| | **MAG (µV*s)** | 6.89±4.24 | 3.99±3.33 | 5.61±5.22 | 5.41±3.64 | 5.42±3.01 | 7.53±4.05 | 7.42±5.37 | 4.14±2.35 | 8.46±8.00 |
| **FP** | **OL (s)** | 0.54±0.32 | 0.58±0.25 | 0.56±0.24 | 0.39±0.11 | 0.40±0.09 | 0.51±0.19 | 0.39±0.09 | 0.52±0.23 | 0.54±0.29 |
| | **DA (s)** | 0.81±0.26 | 0.94±0.34 | 0.96±0.16 | 0.84±0.20 | 1.01±0.29 | 0.97±0.26 | 0.96±0.33 | 0.99±0.27 | 1.09±0.24 |
| | **MAG (µV*s)** | 17.32±9.77 | 28.59±18.22 | 31.77±15.76 | 15.77±9.21 | 28.43±17.73 | 29.89±16.18 | 18.68±11.20 | 28.22±17.97 | 33.44±18.12 |
| **BP** | **OL (s)** | 0.32±0.09 | 0.36±0.14 | 0.46±0.25 | 0.45±0.20 | 0.47±0.24 | 0.44±0.18 | 0.44±0.19 | 0.39±0.18 | 0.39±0.12 |
| | **DA (s)** | 0.98±0.26 | 0.94±0.24 | 0.90±0.33 | 0.98±0.25 | 1.07±0.28 | 1.21±0.26 | 1.16±0.33 | 1.13±0.38 | 1.18±0.25 |
| | **MAG (µV*s)** | 17.02±7.12 | 15.68±5.77 | 18.31±9.96 | 16.27±7.24 | 17.42±6.17 | 21.86±7.43 | 17.61±7.14 | 18.46±7.96 | 22.00±9.49 |
| | | **Paraspinal right** | | | **Biceps femoris left** | | | **Biceps femoris right** | | |

**Legend**. DP, UP, FP and BP represent, respectively, downward, upward, forward and backward perturbations. OL: onset latency; DA: duration of activation; MAG: activation magnitude; DC: dynamic camera; SC: static camera; EC: eyes closed.
Color codes: blue represent shoulder abductors, orange posterior muscles and green anterior muscles.

**Table 1:** Values of EMG parameters during standing conditions

| | | Gastrocnemius left | | | Gastrocnemius right | | | External oblique left | | |
|---|---|---|---|---|---|---|---|---|---|---|
| | | DC | SC | EC | DC | SC | EC | DC | SC | EC |
| DP | OL (s) | 0.97±0.19 | 0.96±0.32 | 0.96±0.24 | 0.93±0.14 | 1.00±0.14 | 0.98±0.09 | 0.97±0.46 | 0.90±0.53 | 0.85±0.41 |
| | DA (s) | 0.88±0.26 | 0.89±0.32 | 0.89±0.21 | 0.99±0.14 | 0.98±0.12 | 0.97±0.07 | 0.80±0.38 | 0.84±0.47 | 0.95±0.35 |
| | MAG (µV*s) | 7.99±3.79 | 6.82±4.05 | 10.53±4.92 | 9.03±4.29 | 8.68±5.77 | 12.76±7.01 | 6.37±4.41 | 6.84±5.86 | 9.84±7.54 |
| UP | OL (s) | 0.72±0.57 | 0.84±0.66 | 0.65±0.45 | 0.67±0.54 | 0.90±0.62 | 0.62±0.40 | 0.99±0.39 | 0.80±0.43 | 0.97±0.48 |
| | DA (s) | 0.80±0.37 | 0.69±0.48 | 1.03±0.43 | 0.63±0.31 | 0.59±0.43 | 0.68±0.25 | 0.75±0.39 | 0.95±0.46 | 0.73±0.39 |
| | MAG (µV*s) | 6.76±6.12 | 4.68±4.64 | 8.75±5.52 | 7.50±6.06 | 5.82±7.98 | 7.57±2.94 | 5.93±5.58 | 4.79±2.71 | 5.90±5.59 |
| FP | OL (s) | 0.57±0.26 | 0.64±0.21 | 0.63±0.21 | 0.57±0.24 | 0.63±0.22 | 0.63±0.20 | 0.33±0.07 | 0.35±0.09 | 0.41±0.18 |
| | DA (s) | 1.05±0.22 | 1.07±0.21 | 1.03±0.23 | 1.05±0.15 | 1.06±0.16 | 1.09±0.17 | 0.93±0.24 | 0.90±0.20 | 0.96±0.28 |
| | MAG (µV*s) | 22.72±11.33 | 32.05±16.07 | 33.81±16.57 | 23.72±11.41 | 32.86±14.93 | 36.27±16.84 | 15.57±6.63 | 22.61±14.34 | 27.76±16.85 |
| BP | OL (s) | 0.39±0.23 | 0.30±0.04 | 0.31±0.14 | 0.32±0.11 | 0.34±0.10 | 0.37±0.13 | 0.80±0.38 | 0.77±0.29 | 0.84±0.30 |
| | DA (s) | 0.87±0.30 | 0.95±0.32 | 1.01±0.31 | 1.01±0.30 | 0.95±0.33 | 1.11±0.34 | 0.92±0.32 | 0.91±0.21 | 0.89±0.28 |
| | MAG (µV*s) | 15.73±7.26 | 18.50±7.59 | 21.99±10.31 | 18.79±7.90 | 18.93±8.14 | 23.61±10.37 | 12.28±6.72 | 13.41±7.00 | 14.34±10.19 |

**Legend**. DP, UP, FP and BP represent, respectively, downward, upward, forward and backward perturbations. OL: onset latency; DA: duration of activation; MAG: activation magnitude; DC: dynamic camera; SC: static camera; EC: eyes closed.
Color codes: blue represent shoulder abductors, orange posterior muscles and green anterior muscles.

**Table 1:** Values of EMG parameters during standing conditions

| | | DC | SC | EC | DC | SC | EC | DC | SC | EC |
|---|---|---|---|---|---|---|---|---|---|---|
| **DP** | **OL (s)** | 0.88±0.34 | 0.99±0.57 | 0.82±0.40 | 1.02±0.39 | 0.92±0.51 | 0.91±0.36 | 0.92±0.43 | 0.74±0.39 | 0.94±0.39 |
| | **DA (s)** | 0.79±0.26 | 0.72±0.45 | 0.86±0.43 | 0.66±0.30 | 0.73±0.39 | 0.64±0.32 | 0.64±0.30 | 0.74±0.30 | 0.62±0.37 |
| | **MAG (µV*s)** | 5.44±2.72 | 4.55±4.09 | 8.49±6.36 | 4.92±3.22 | 5.19±5.21 | 6.16±2.12 | 4.10±2.09 | 4.47±2.89 | 6.42±6.47 |
| **UP** | **OL (s)** | 0.96±0.37 | 0.76±0.38 | 1.05±0.31 | 0.97±0.46 | 0.97±0.51 | 1.04±0.52 | 0.96±0.40 | 1.08±0.49 | 1.12±0.31 |
| | **DA (s)** | 0.76±0.36 | 0.93±0.26 | 0.82±0.35 | 0.64±0.32 | 0.72±0.34 | 0.56±0.37 | 0.65±0.31 | 0.58±0.31 | 0.64±0.18 |
| | **MAG (µV*s)** | 5.54±5.89 | 4.77±2.92 | 5.05±3.47 | 4.55±3.87 | 3.48±2.60 | 3.91±3.54 | 4.75±5.60 | 2.36±1.87 | 4.21±3.01 |
| **FP** | **OL (s)** | 0.35±0.08 | 0.37±0.08 | 0.45±0.18 | 0.36±0.09 | 0.44±0.08 | 0.45±0.21 | 0.35±0.12 | 0.38±0.10 | 0.41±0.17 |
| | **DA (s)** | 0.83±0.26 | 0.81±0.22 | 0.79±0.19 | 0.65±0.24 | 0.66±0.19 | 0.69±0.27 | 0.68±0.25 | 0.69±0.23 | 0.66±0.21 |
| | **MAG (µV*s)** | 13.21±6.94 | 18.84±11.69 | 22.30±13.37 | 10.55±6.77 | 16.01±13.64 | 17.42±12.89 | 10.33±6.85 | 15.85±12.08 | 16.69±9.39 |
| **BP** | **OL (s)** | 0.92±0.42 | 0.95±0.29 | 0.98±0.38 | 0.91±0.34 | 1.00±0.32 | 0.84±0.23 | 0.95±0.54 | 0.97±0.39 | 0.88±0.18 |
| | **DA (s)** | 0.83±0.30 | 0.74±0.29 | 0.79±0.38 | 0.78±0.31 | 0.59±0.25 | 0.84±0.24 | 0.66±0.40 | 0.66±0.32 | 0.73±0.25 |
| | **MAG (µV*s)** | 11.73±9.29 | 9.89±6.75 | 12.90±10.18 | 10.18±5.72 | 8.25±5.45 | 14.62±9.25 | 9.91±8.94 | 8.85±5.60 | 12.68±9.62 |
| | | **External oblique right** | | | **Rectus abdominis left** | | | **Rectus abdominis right** | | |

**Legend**. DP, UP, FP and BP represent, respectively, downward, upward, forward and backward perturbations. OL: onset latency; DA: duration of activation; MAG: activation magnitude; DC: dynamic camera; SC: static camera; EC: eyes closed.
Color codes: blue represent shoulder abductors, orange posterior muscles and green anterior muscles.

**Table 1:** Values of EMG parameters during standing conditions

| | | Rectus femoris left | | | Rectus femoris right | | | Tibialis anterior left | | |
|---|---|---|---|---|---|---|---|---|---|---|
| | | DC | SC | EC | DC | SC | EC | DC | SC | EC |
| DP | OL (s) | 0.81±0.46 | 1.06±0.37 | 0.66±0.35 | 0.77±0.33 | 0.85±0.57 | 0.66±0.32 | 0.89±0.19 | 0.92±0.19 | 0.86±0.21 |
| | DA (s) | 0.79±0.39 | 0.67±0.36 | 0.85±0.27 | 0.70±0.35 | 0.74±0.44 | 0.89±0.18 | 0.87±0.22 | 0.93±0.25 | 0.95±0.15 |
| | MAG (µV*s) | 6.16±4.44 | 4.34±2.69 | 8.62±6.53 | 4.81±3.39 | 5.88±7.10 | 7.95±3.59 | 7.37±4.08 | 7.55±4.56 | 10.93±6.20 |
| UP | OL (s) | 0.74±0.38 | 1.07±0.38 | 0.84±0.36 | 0.93±0.43 | 0.98±0.33 | 0.93±0.35 | 0.81±0.46 | 1.10±0.45 | 0.90±0.35 |
| | DA (s) | 0.85±0.33 | 0.64±0.32 | 0.79±0.30 | 0.78±0.34 | 0.75±0.27 | 0.72±0.32 | 0.81±0.32 | 0.65±0.21 | 0.73±0.25 |
| | MAG (µV*s) | 6.43±4.94 | 3.08±2.13 | 5.21±2.74 | 5.23±3.58 | 3.62±2.25 | 4.03±1.71 | 6.07±3.75 | 3.57±2.34 | 5.59±2.33 |
| FP | OL (s) | 0.36±0.03 | 0.37±0.06 | 0.39±0.11 | 0.34±0.03 | 0.35±0.05 | 0.37±0.12 | 0.35±0.07 | 0.39±0.15 | 0.35±0.08 |
| | DA (s) | 0.76±0.14 | 0.93±0.24 | 1.03±0.32 | 0.88±0.30 | 0.90±0.21 | 0.94±0.38 | 0.84±0.31 | 0.93±0.32 | 1.02±0.33 |
| | MAG (µV*s) | 11.96±6.65 | 23.95±18.52 | 27.62±17.66 | 14.01±7.85 | 22.76±14.79 | 25.90±18.81 | 15.17±10.58 | 25.45±18.82 | 28.61±18.63 |
| BP | OL (s) | 0.64±0.25 | 0.82±0.17 | 0.79±0.19 | 0.73±0.26 | 0.84±0.18 | 0.82±0.18 | 0.45±0.22 | 0.54±0.27 | 0.49±0.23 |
| | DA (s) | 1.01±0.28 | 0.92±0.21 | 0.98±0.23 | 0.95±0.17 | 0.90±0.22 | 0.92±0.15 | 0.95±0.39 | 0.90±0.35 | 1.10±0.25 |
| | MAG (µV*s) | 14.42±6.84 | 11.24±4.64 | 14.62±7.67 | 12.69±6.62 | 10.36±5.68 | 13.85±7.28 | 15.74±9.18 | 14.08±5.91 | 19.25±9.44 |

**Legend**. DP, UP, FP and BP represent, respectively, downward, upward, forward and backward perturbations. OL: onset latency; DA: duration of activation; MAG: activation magnitude; DC: dynamic camera; SC: static camera; EC: eyes closed.
Color codes: blue represent shoulder abductors, orange posterior muscles and green anterior muscles.

**Table 1:** Values of EMG parameters during standing conditions

| | | DC | SC | EC |
|---|---|---|---|---|
| DP | OL (s) | 0.83±0.21 | 1.03±0.22 | 0.92±0.16 |
| | DA (s) | 0.98±0.20 | 0.81±0.22 | 0.94±0.20 |
| | MAG (μV*s) | 8.49±4.24 | 7.00±3.98 | 11.55±6.71 |
| UP | OL (s) | 0.83±0.61 | 0.93±0.65 | 0.79±0.38 |
| | DA (s) | 0.73±0.47 | 0.60±0.41 | 0.65±0.22 |
| | MAG (μV*s) | 6.74±5.98 | 4.02±3.74 | 6.55±4.95 |
| FP | OL (s) | 0.33±0.03 | 0.38±0.17 | 0.34±0.07 |
| | DA (s) | 0.74±0.22 | 0.92±0.31 | 0.99±0.31 |
| | MAG (μV*s) | 14.64±10.72 | 24.66±18.00 | 27.52±17.75 |
| BP | OL (s) | 0.49±0.25 | 0.62±0.24 | 0.60±0.27 |
| | DA (s) | 0.88±0.21 | 0.87±0.31 | 1.03±0.34 |
| | MAG (μV*s) | 14.61±7.38 | 13.29±7.20 | 17.42±11.22 |
| | | **Tibialis anterior right** | | |

**Legend**. DP, UP, FP and BP represent, respectively, downward, upward, forward and backward perturbations. OL: onset latency; DA: duration of activation; MAG: activation magnitude; DC: dynamic camera; SC: static camera; EC: eyes closed.
Color codes: blue represent shoulder abductors, orange posterior muscles and green anterior muscles.
