# Supplementary material for: Patterns of whole-body muscle activations following vertical perturbations during standing and walking
Source: J Neuroeng Rehabil. 2021 May 6;18:75. doi: 10.1186/s12984-021-00836-0 (PMC8101216; doi:10.1186/s12984-021-00836-0)
Supplement: Supplementary file 4 — Additional file 4: Table S2. Values of EMG parameters during walking conditions. [file 12984_2021_836_MOESM4_ESM.pdf]

Table 2: Values of EMG parameters during walking conditions

		Deltoid		Paraspinals		Biceps femoris		Gastrocnemius	
		ipsilateral	contralateral	ipsilateral	contralateral	ipsilateral	contralateral	ipsilateral	contralateral
**DP**	**OL (s)**	0.55±0.36	0.38±0.33	0.53±0.35	0.41±0.32	0.44±0.33	0.47±0.36	0.50±0.32	0.95±0.52
	**DA (s)**	0.79±0.29	0.95±0.31	0.80±0.33	0.95±0.34	0.79±0.28	0.79±0.28	0.84±0.35	0.53±0.29
	**MAG (µV*s)**	39.85±25.85	49.56±29.84	42.68±36.99	48.71±31.57	46.30±26.29	40.20±20.81	51.14±39.12	38.93±30.95
**UP**	**OL (s)**	0.48±0.30	0.52±0.39	0.41±0.22	0.47±0.34	0.38±0.25	0.45±0.31	0.55±0.28	0.60±0.31
	**DA (s)**	0.83±0.31	0.93±0.30	1.00±0.28	0.97±0.29	0.89±0.27	0.87±0.30	0.84±0.27	0.67±0.25
	**MAG (µV*s)**	56.42±37.10	61.44±42.24	66.46±44.72	65.12±44.83	61.67±40.43	59.10±39.89	61.81±38.89	51.41±35.85
**FP**	**OL (s)**	0.36±0.18	0.46±0.25	0.46±0.18	0.37±0.19	0.56±0.36	0.37±0.17	0.56±0.27	0.57±0.25
	**DA (s)**	0.99±0.27	0.94±0.24	0.99±0.23	1.02±0.25	0.83±0.28	0.86±0.25	0.84±0.27	0.91±0.29
	**MAG (µV*s)**	66.95±27.62	69.66±37.90	74.42±44.02	78.27±45.67	69.64±38.46	68.05±35.92	72.15±41.39	74.78±43.46
**BP**	**OL (s)**	0.43±0.31	0.41±0.33	0.49±0.43	0.44±0.39	0.41±0.31	0.34±0.16	0.40±0.31	0.88±0.60
	**DA (s)**	0.81±0.28	0.85±031	0.70±0.26	0.80±0.29	0.75±0.28	0.77±0.27	0.82±0.34	0.65±0.35
	**MAG (µV*s)**	48.23±28.00	51.11±31.75	47.23±32.87	50.35±38.50	49.91±32.00	49.29±26.34	57.62±38.47	50.14±42.84

Legend. DP, UP, FP and BP represent, respectively, downward, upward, forward and backward perturbations. OL: onset latency; DA: duration of activation; MAG: activation magnitude. Color codes: blue represent shoulder abductors, orange posterior muscles and green anterior muscles. Opaque, clear colors respectively indicate contralateral, ipsilateral side.

Table 2: Values of EMG parameters during walking conditions

		External oblique		Rectus abdominis		Rectus femoris		Tibialis anterior	
		ipsilateral	contralateral	ipsilateral	contralateral	ipsilateral	contralateral	ipsilateral	contralateral
**DP**	**OL (s)**	0.52±0.34	0.40±0.37	0.58±0.36	0.52±0.37	0.50±0.39	0.44±0.26	0.62±0.39	0.49±0.30
	**DA (s)**	0.79±0.32	0.93±0.39	0.78±0.29	0.79±0.32	0.85±0.35	0.79±0.23	0.89±0.33	0.78±0.27
	**MAG (µV*s)**	35.45±23.10	45.48±22.03	35.46±22.25	35.51±19.48	44.34±29.07	37.34±19.93	48.66±33.27	43.33±34.90
**UP**	**OL (s)**	0.53±0.34	0.42±0.34	0.51±0.26	0.37±0.25	0.36±0.26	0.34±0.16	0.41±0.21	0.50±0.24
	**DA (s)**	0.77±0.28	0.90±0.34	0.86±0.22	0.85±0.34	0.90±0.29	0.87±0.22	0.99±0.32	0.90±0.33
	**MAG (µV*s)**	50.13±30.36	57.45±41.01	58.25±42.34	56.79±38.03	57.46±30.80	63.90±49.83	67.88±48.10	62.40±41.13
**FP**	**OL (s)**	0.53±0.32	0.33±0.19	0.48±0.19	0.42±0.20	0.39±0.21	0.45±0.23	0.33±0.19	0.44±0.19
	**DA (s)**	0.82±0.28	0.99±0.28	0.90±0.26	0.90±0.24	0.89±0.20	0.88±0.18	1.02±0.25	1.11±0.28
	**MAG (µV*s)**	65.00±45.36	72.41±41.06	67.77±44.45	61.26±26.64	65.81±28.18	67.84±38.92	81.35±57.82	79.89±37.82
**BP**	**OL (s)**	0.44±0.29	0.28±0.22	0.38±0.26	0.38±0.29	0.44±0.35	0.32±0.15	0.45±0.28	0.39±0.19
	**DA (s)**	0.76±0.27	0.89±0.24	0.81±0.23	0.84±0.29	0.78±0.29	0.83±0.20	0.94±0.28	0.80±0.26
	**MAG (µV*s)**	48.92±26.28	51.50±30.07	51.22±26.74	55.53±37.89	42.20±22.21	48.81±23.42	56.57±35.40	51.74±32.88

Legend. DP, UP, FP and BP represent, respectively, downward, upward, forward and backward perturbations. OL: onset latency; DA: duration of activation; MAG: activation magnitude. Color codes: blue represent shoulder abductors, orange posterior muscles and green anterior muscles. Opaque, clear colors respectively indicate contralateral, ipsilateral side.
