# Supplementary material for: Patterns of whole-body muscle activations following vertical perturbations during standing and walking
Source: J Neuroeng Rehabil. 2021 May 6;18:75. doi: 10.1186/s12984-021-00836-0 (PMC8101216; doi:10.1186/s12984-021-00836-0)
Supplement: Supplementary file 5 — Additional file 5: Table S3. Values of EMG parameters after visual perturbations. [file 12984_2021_836_MOESM5_ESM.pdf]

Table 3: Values of EMG parameters after visual perturbations

		Deltoid		Paraspinals		Biceps femoris		Gastrocnemius	
		Left	Right	Left	Right	Left	Right	Left	Right
DP	OL (s)	0.70±0.37	0.84±0.43	1.06±0.47	1.14±0.41	0.95±0.42	0.82±0.62	0.97±0.43	1.04±0.34
	DA (s)	0.83±0.47	0.81±0.27	0.65±0.38	0.58±0.39	0.69±0.31	0.86±0.47	0.82±0.33	0.73±0.34
	MAG (µV*s)	4.37±3.73	3.76±1.76	3.62±4.19	3.60±4.87	3.67±2.55	3.91±3.46	4.88±3.87	5.67±5.77
UP	OL (s)	0.91±0.41	1.02±0.60	1.06±0.45	1.13±0.55	0.75±0.56	0.81±0.57	1.02±0.50	0.67±0.32
	DA (s)	0.66±0.38	0.68±0.41	0.59±0.40	0.59±0.33	0.89±0.47	0.84±0.48	0.78±0.45	0.98±0.33
	MAG (µV*s)	3.33±3.12	2.86±2.23	2.71±1.78	2.87±2.37	4.32±3.87	6.03±6.79	5.03±5.49	6.74±4.86
FP	OL (s)	1.17±0.46	0.89±0.33	1.18±0.44	1.10±0.53	1.12±0.43	1.18±0.22	1.27±0.34	1.32±0.30
	DA (s)	0.56±0.30	0.69±0.28	0.60±0.31	0.54±0.35	0.70±0.34	0.61±0.22	0.62±0.30	0.53±0.24
	MAG (µV*s)	3.71±3.44	2.70±1.53	3.11±2.55	2.97±2.50	3.71±3.76	3.76±3.18	3.80±2.48	4.93±6.80
BP	OL (s)	1.09±0.44	0.82±0.50	1.11±0.44	0.79±0.56	0.96±0.35	0.89±0.30	0.88±0.34	0.83±0.28
	DA (s)	0.62±0.42	0.86±0.31	0.64±0.37	0.92±0.53	0.77±0.29	0.81±0.25	0.86±0.31	0.92±0.22
	MAG (µV*s)	3.25±2.77	4.30±2.65	3.02±2.16	4.40±3.84	5.33±5.57	4.39±3.06	5.67±4.61	6.14±4.40

Legend. DP, UP, FP and BP represent, respectively, downward, upward, forward and backward perturbations.
OL: onset latency; DA: duration of activation; MAG: activation magnitude;
Color codes: blue represent shoulder abductors, orange posterior muscles and green anterior muscles.

Table 3: Values of EMG parameters after visual perturbations

		External oblique		Rectus abdominis		Rectus femoris		Tibialis anterior	
		Left	Right	Left	Right	Left	Right	Left	Right
DP	OL (s)	1.06±0.40	1.12±0.37	1.30±0.43	1.08±0.41	0.78±0.38	1.00±0.61	0.93±0.42	0.84±0.48
	DA (s)	0.66±0.33	0.66±0.36	0.44±0.32	0.55±0.25	0.88±0.26	0.82±0.52	0.83±0.42	0.98±0.44
	MAG (µV*s)	3.10±1.77	3.23±1.92	2.50±1.93	2.88±1.92	4.19±2.13	3.56±2.19	4.47±0.31	7.22±6.75
UP	OL (s)	1.12±0.40	1.09±0.33	1.11±0.42	1.11±0.56	0.83±0.30	1.09±0.42	1.15±0.55	1.18±0.38
	DA (s)	0.60±0.37	0.71±0.29	0.54±0.29	0.51±0.30	0.72±0.29	0.62±0.29	0.64±0.43	0.60±0.24
	MAG (µV*s)	3.65±3.71	3.29±2.19	2.53±2.25	2.50±2.45	4.29±3.69	3.24±3.05	3.14±2.68	3.25±2.30
FP	OL (s)	1.33±0.39	1.14±0.42	1.15±0.36	0.98±0.47	1.21±0.51	0.88±0.37	1.07±0.35	1.26±0.27
	DA (s)	0.45±0.37	0.63±0.36	0.50±0.33	0.72±0.36	0.61±0.44	0.83±0.42	0.71±0.39	0.60±0.23
	MAG (µV*s)	1.95±1.94	3.52±4.36	2.00±1.28	2.63±1.43	2.58±2.15	4.07±2.71	4.06±3.92	3.63±3.47
BP	OL (s)	0.99±0.35	0.97±0.48	1.03±0.36	0.84±0.43	0.92±0.47	1.01±0.46	0.73±0.36	0.91±0.38
	DA (s)	0.73±0.34	0.86±0.48	0.62±0.39	0.84±0.50	0.77±0.45	0.67±0.40	0.95±0.38	0.87±0.32
	MAG (µV*s)	3.69±4.35	4.36±3.00	3.48±3.54	4.61±3.88	3.44±2.60	2.98±2.73	5.26±5.39	4.61±3.03

Legend. DP, UP, FP and BP represent, respectively, downward, upward, forward and backward perturbations.
OL: onset latency; DA: duration of activation; MAG: activation magnitude;
Color codes: blue represent shoulder abductors, orange posterior muscles and green anterior muscles.
