# Supplementary material for: Patterns of whole-body muscle activations following vertical perturbations during standing and walking
Source: J Neuroeng Rehabil. 2021 May 6;18:75. doi: 10.1186/s12984-021-00836-0 (PMC8101216; doi:10.1186/s12984-021-00836-0)

**SUPPLEMENTARY FILE #6**

***Entire ANOVA table outputs from the statistical analysis of onset latency and duration of activation in standing and walking conditions***

From statistical analysis of standing conditions

Statistical output from the three-way analysis of *onset latency*, including muscle, perturbation direction and sensory condition as factors.


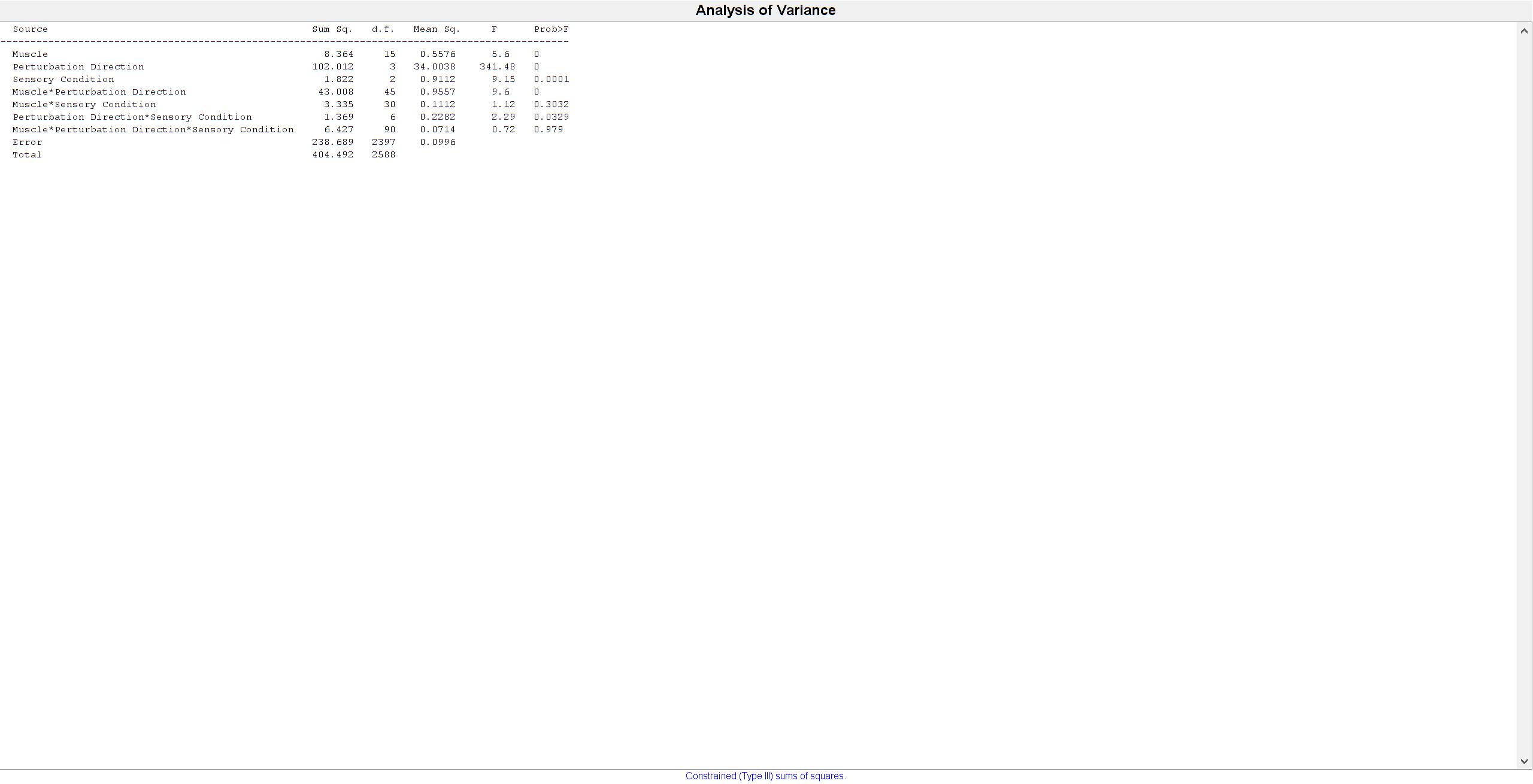


Statistical output from the three-way analysis of *duration of activation*, including muscle, perturbation direction and sensory condition as factors.


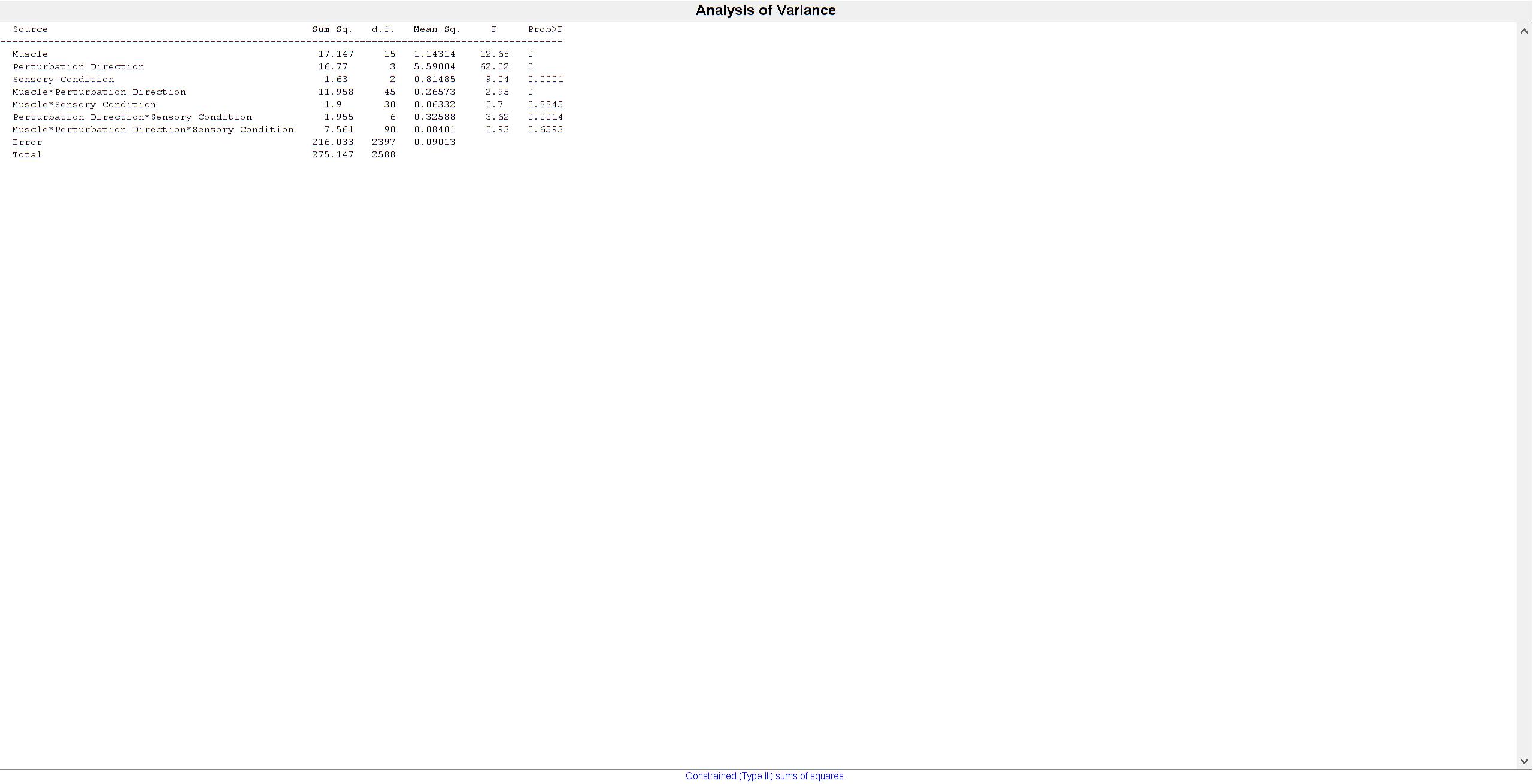


From statistical analysis of walking conditions

Statistical output from the three-way analysis of *onset latency*, including muscle, perturbation direction and perturbation side as factors.


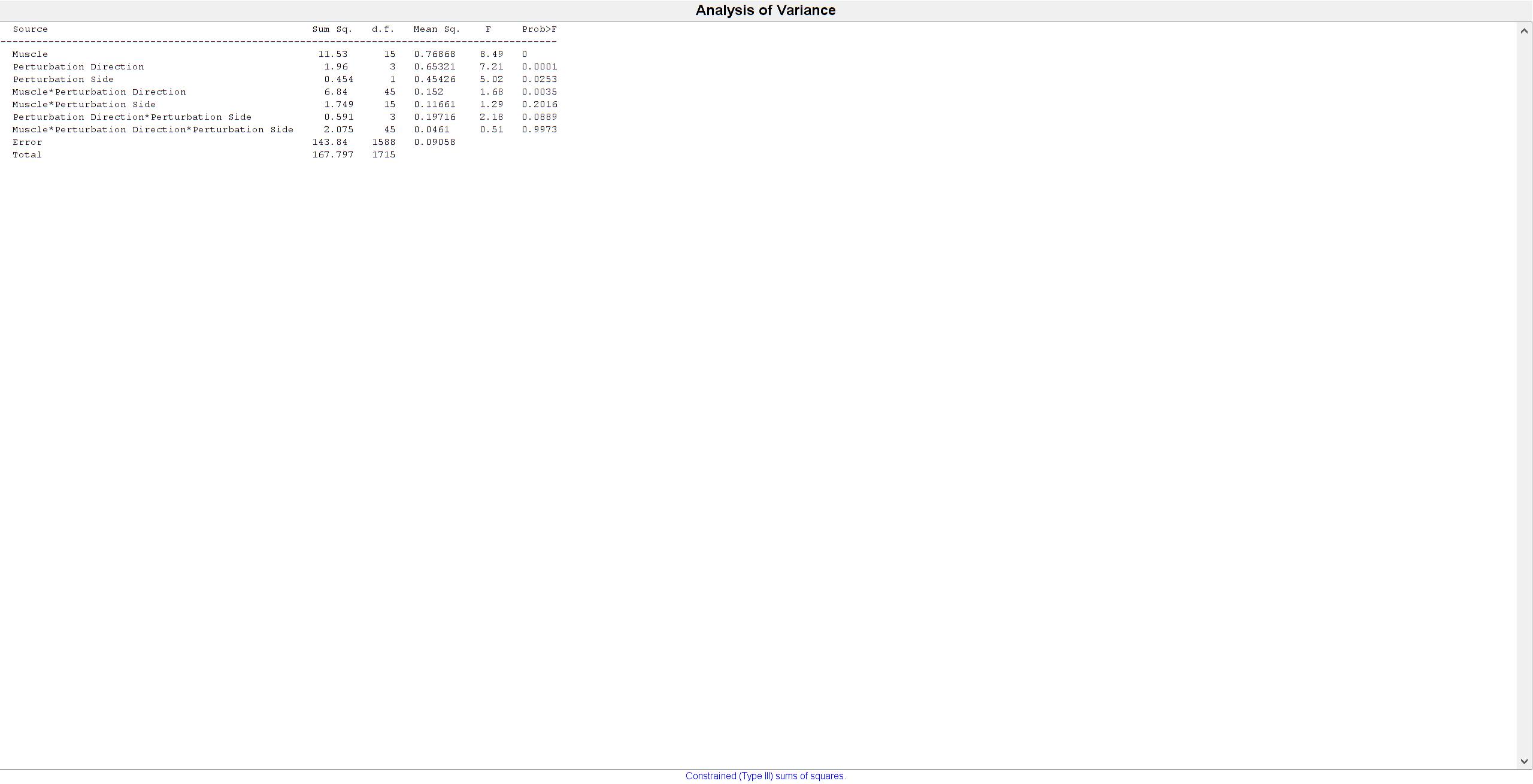


Statistical output from the three-way analysis of *duration of activation*, including muscle, perturbation direction and perturbation side as factors.


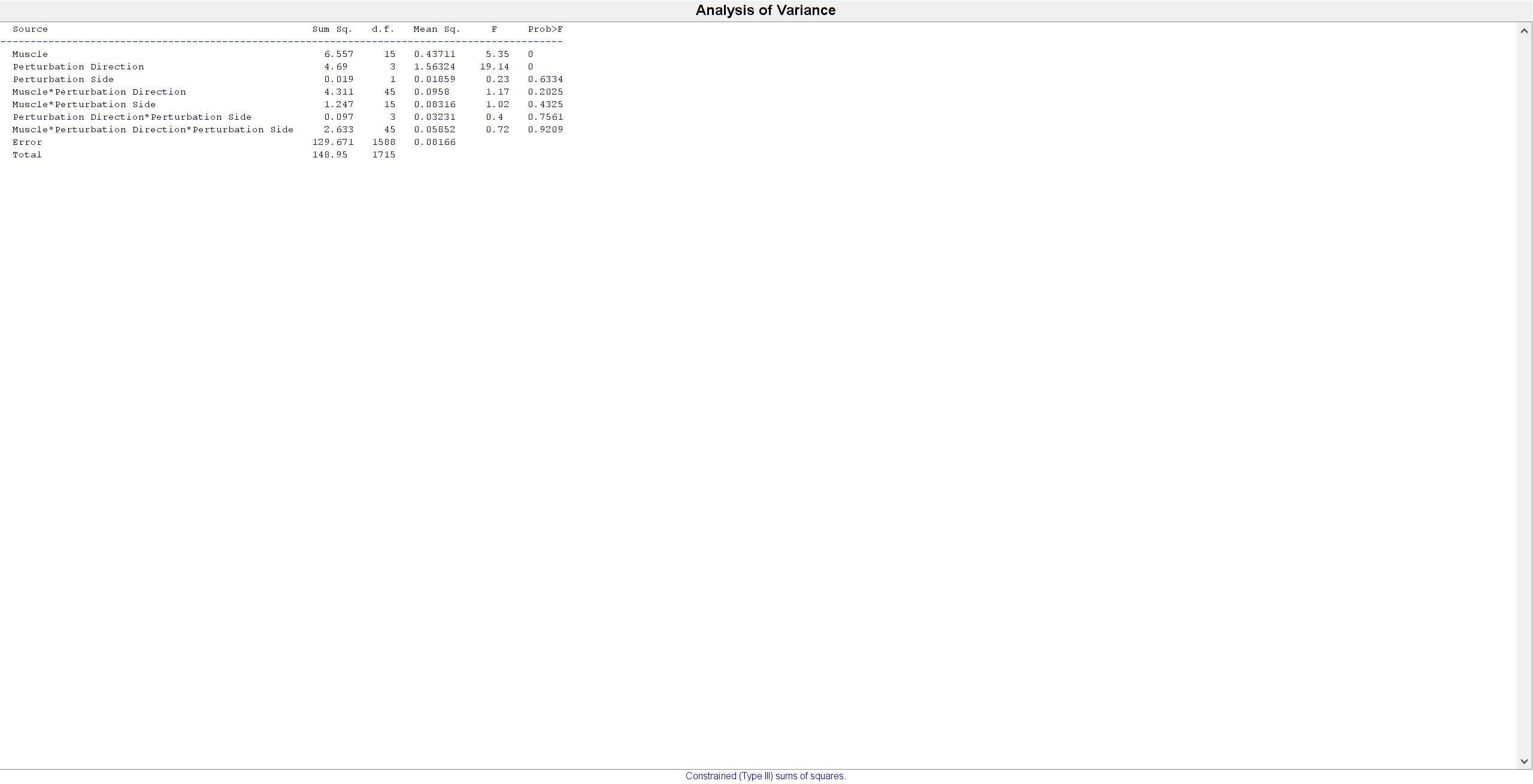


From statistical analysis of visual conditions

Statistical output from the two-way analysis of *onset latency*, including muscle and perturbation direction as factors.


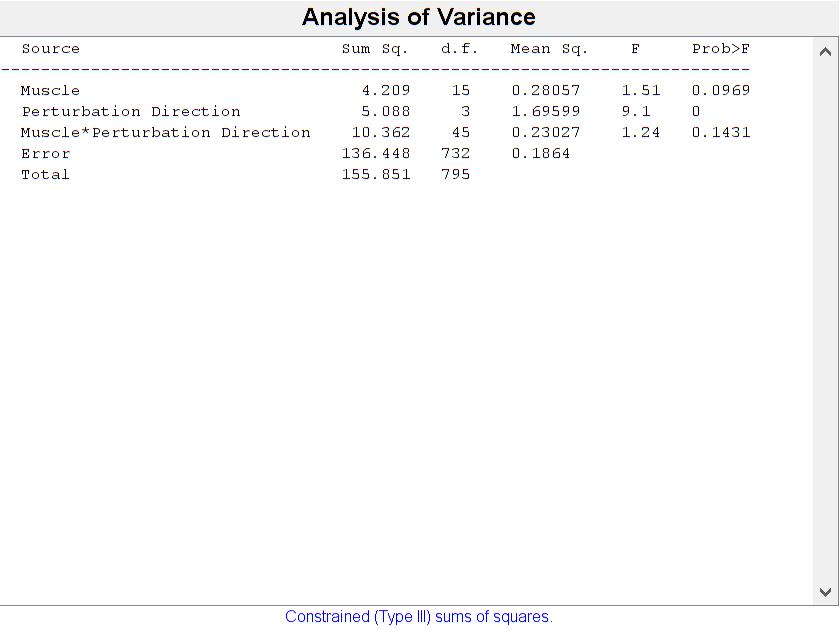


Statistical output from the two-way analysis of *duration of activation*, including muscle and perturbation direction as factors.


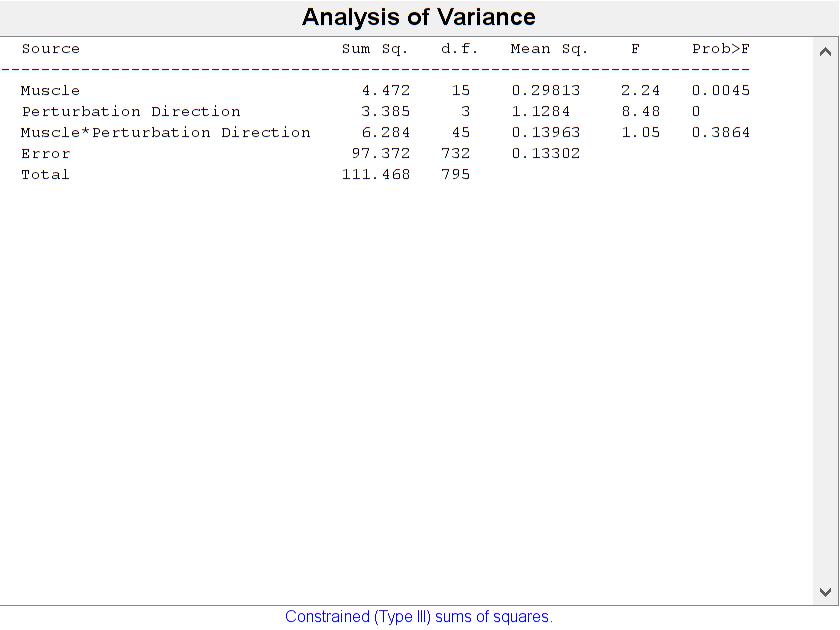

Supplement: Supplementary file 6 — Additional file 6: Entire ANOVA table outputs from the statistical analysis of onset latency and duration of activation in standing and walking conditions. [file 12984_2021_836_MOESM6_ESM.docx]
